# Supplementary material for: A complex epistatic network limits the mutational reversibility in the influenza hemagglutinin receptor-binding site
Source: Nat Commun. 2018 Mar 28;9:1264. doi: 10.1038/s41467-018-03663-5 (PMC5871881; doi:10.1038/s41467-018-03663-5)
Supplement: Supplementary file 1 — Supplementary Information(PDF 14823 kb) [file 41467_2018_3663_MOESM1_ESM.pdf]

**Supplementary Table 1.** Amino-acid identities at residues of interest.

| | Residues | | | | | | | | | | | | | | | | |
|---|---|---|---|---|---|---|---|---|---|---|---|---|---|---|---|---|---|
| Strains | 145 | 155 | 156 | 159 | 160 | 186 | 189 | 190 | 192 | 193 | 194 | 196 | 202 | 219 | 222 | 225 | 227 |
| A/HongKong/1/1968 | S | T | K | S | T | S | Q | E | T | S | L | V | V | S | W | G | S |
| A/Shangdong/9/1993 | N | H | K | Y | K | S | S | D | T | S | L | V | V | S | W | G | S |
| A/Moscow/10/1999 | N | H | Q | Y | R | S | S | D | T | S | L | T | V | S | W | G | S |
| A/Wyoming/3/2003 | K | T | H | Y | K | V | S | D | I | S | L | A | I | F | R | D | S |
| A/Finland/486/2004 | N | T | H | L | K | G | N | D | I | S | L | A | I | S | R | D | P |
| A/Hong Kong/4443/2005 | N | T | H | L | K | G | N | D | I | F | L | A | I | S | R | N | P |
| A/Brisbane/10/2007 | N | T | H | F | K | G | N | D | I | F | P | A | I | S | R | N | P |
| A/Perth/16/2009 | N | T | H | F | K | G | K | D | I | F | L | A | I | S | R | N | P |
| A/Victoria/361/2011 | N | T | H | F | K | G | K | D | I | F | L | A | I | S | R | N | P |
| A/Michigan/15/2014 | S | T | H | Y | K | G | K | D | I | F | L | A | I | S | R | D | P |
| A/North Dakota/26/2016 | S | T | H | S | K | G | K | D | I | F | L | A | I | S | R | D | P |

**Supplementary Table 2.** X-ray data collection and refinement statistics.

| **Data collection** | HK68 E190D Apo | Wy03 Apo | Wy03 D190E Apo | Mich14 Apo | HK68 E190D + 6'-SLN | Wy03 + 6'-SLN | Wy03 D190E + 6'-SLN | Mich14 + 6'-SLN |
|---|---|---|---|---|---|---|---|---|
| Beamline | APS 23ID-D | SSRL 12-2 | APS 23ID-B | SSRL 12-2 | APS 23ID-D | APS 23ID-B | APS 23ID-B | SSRL 12-2 |
| Wavelength (Å) | 1.0332 | 0.9795 | 1.0332 | 0.9795 | 1.0332 | 1.0332 | 1.0332 | 0.9795 |
| Space group | C2 | H32 | H32 | H32 | C2 | H32 | H32 | H32 |
| Unit cell parameters (Å and °) | a=209.2, b=131.4, c=71.9, β=97.9 | a=b=100.3, c=385.6 | a=b=100.4, c=384.4 | a=b=100.3, c=394.1 | a=208.8, b=130.8, c=72.2, β=97.9 | a=b=100.5, c=385.7 | a=b=100.6, c=385.6 | a=b=100.4, c=394.3 |
| Resolution (Å) | 50-2.20 (2.28-2.20)[a] | 50-1.85 (1.91-1.85)[a] | 50-1.85 (1.91-1.85)[a] | 50-2.05 (2.12-2.05)[a] | 50-2.25 (2.32-2.25)[a] | 50-1.76 (1.82-1.76)[a] | 50-1.80 (1.86-1.80)[a] | 50-1.80 (1.86-1.80)[a] |
| Unique Reflections | 96,961 (9,442)[a] | 63,929 (5,808)[a] | 64,323 (5,731)[a] | 48,747 (4,806)[a] | 91,326 (8,165)[a] | 74,408 (6,203)[a] | 70,033 (6,052)[a] | 71,063 (7,073)[a] |
| Redundancy | 4.5 (3.2)[a] | 17.5 (16.0)[a] | 18.0 (14.0)[a] | 17.4 (16.0)[a] | 6.5 (4.5)[a] | 18.0 (14.5)[a] | 17.1 (11.8)[a] | 7.3 (7.6)[a] |
| Completeness (%) | 99.4 (97.2)[a] | 98.7 (99.5)[a] | 100.0 (99.6)[a] | 100.0 (100.0)[a] | 99.8 (98.2)[a] | 99.3 (92.5)[a] | 99.6 (95.5)[a] | 99.1 (100.0)[a] |
| $<I/\sigma_I>$ | 16.3 (1.2)[a] | 43.3 (2.7)[a] | 36.7 (2.2)[a] | 44.3 (3.1)[a] | 15.7 (1.5)[a] | 52.0 (3.6)[a] | 39.9 (2.5)[a] | 33.0 (1.6)[a] |
| $R_{sym}$[b] | 0.10 (0.71)[a] | 0.09 (0.95)[a] | 0.08 (0.65)[a] | 0.15 (0.92)[a] | 0.08 (0.59)[a] | 0.09 (0.48)[a] | 0.08 (0.45)[a] | 0.06 (0.93)[a] |
| $R_{pim}$[b] | 0.05 (0.44)[a] | 0.02 (0.24)[a] | 0.02 (0.17)[a] | 0.04 (0.24)[a] | 0.04 (0.30)[a] | 0.02 (0.13)[a] | 0.02 (0.12)[a] | 0.02 (0.36)[a] |
| $CC_{1/2}$[c] | 1.00 (0.71)[a] | 1.00 (0.93)[a] | 1.00 (0.91)[a] | 1.00 (0.91)[a] | 1.00 (0.74)[a] | 1.00 (0.95)[a] | 1.00 (0.95)[a] | 1.00 (0.87)[a] |
| $Z_a$[d] | 3 | 1 | 1 | 1 | 3 | 1 | 1 | 1 |
| **Refinement statistics** | | | | | | | | |
| Resolution (Å) | 50-2.20 | 50-1.85 | 50-1.85 | 50-2.05 | 50-2.25 | 50-1.76 | 50-1.80 | 50-1.80 |
| Reflections (work) | 92,139 | 60,707 | 61,142 | 46,272 | 86,754 | 70,663 | 66,368 | 67,318 |
| Reflections (test) | 4,791 | 3,221 | 3,058 | 2,425 | 4,571 | 3,743 | 3,484 | 3,474 |
| $R_{cryst}$(%)[e] / $R_{free}$(%)[f] | 18.1 / 20.9 | 16.7 / 18.5 | 16.5 / 18.8 | 19.2 / 22.2 | 17.8 / 20.8 | 15.9 / 18.3 | 16.5 / 18.9 | 17.4 / 20.0 |
| No. of atoms | | | | | | | | |
| Protein | 11,523 | 3,918 | 3,912 | 3,899 | 11,517 | 3,928 | 3,920 | 3,941 |
| Water | 698 | 440 | 478 | 310 | 805 | 543 | 523 | 474 |
| Glycan | 318 | 209 | 212 | 137 | 340 | 209 | 212 | 154 |
| 6'-SLN | - | - | - | - | 117 | 46 | 21 | 46 |
| Solvent[g] | 11 | - | - | - | 22 | - | - | 50 |
| Average *B*-value (Å$^2$) | | | | | | | | |
| Protein | 49 | 38 | 31 | 51 | 49 | 31 | 31 | 40 |
| Water | 46 | 48 | 41 | 52 | 44 | 42 | 42 | 50 |
| Glycan | 74 | 63 | 58 | 79 | 74 | 56 | 59 | 69 |
| 6'-SLN | - | - | - | - | 70 | 56 | 57 | 55 |
| Solvent[g] | 72 | - | - | - | 77 | - | - | 63 |
| Wilson *B*-value (Å$^2$) | 37 | 28 | 22 | 38 | 34 | 21 | 21 | 30 |
| **RMSD from ideal geometry** | | | | | | | | |
| Bond length (Å) | 0.010 | 0.011 | 0.012 | 0.010 | 0.010 | 0.011 | 0.012 | 0.012 |
| Bond angle (°) | 1.46 | 1.47 | 1.56 | 1.48 | 1.47 | 1.53 | 1.62 | 1.53 |
| **Ramachandran statistics (%)** | | | | | | | | |
| Favored | 97.1 | 96.8 | 96.8 | 96.8 | 97.3 | 97.2 | 97.0 | 96.4 |
| Outliers | 0.2 | 0.0 | 0.0 | 0.0 | 0.2 | 0.0 | 0.0 | 0.2 |
| **PDB code** | 6BKM | 6BKN | 6BKO | 6BKP | 6BKQ | 6BKR | 6BKS | 6BKT |

[a] Numbers in parentheses refer to the highest resolution shell.

[b] $R_{sym} = \Sigma_{hkl} \Sigma_i | I_{hkl,i} - <I_{hkl}> | / \Sigma_{hkl} \Sigma_i I_{hkl,i}$ and $R_{pim} = \Sigma_{hkl} (1/(n-1))^{1/2} \Sigma_i | I_{hkl,i} - <I_{hkl}> | / \Sigma_{hkl} \Sigma_i I_{hkl,i}$, where $I_{hkl,i}$ is the scaled intensity of the i$^{th}$ measurement of reflection h, k, l, $<I_{hkl}>$ is the average intensity for that reflection, and *n* is the redundancy.

[c] $CC_{1/2}$ = Pearson correlation coefficient between two random half datasets.

[d] $Z_a$ is the number of HA protomers per crystallographic asymmetric unit.

[e] $R_{cryst} = \Sigma_{hkl} | F_o - F_c | / \Sigma_{hkl} | F_o | \times 100$, where $F_o$ and $F_c$ are the observed and calculated structure factors, respectively.

[f] $R_{free}$ was calculated as for $R_{cryst}$, but on a test set comprising 5% of the data excluded from refinement.

[g] Solvent includes Tris, PEG, and sulfate ion.

**Supplementary Table 3.** Index sequence for next-generation sequencing.

| Sample | Index |
|---|---|
| Bris07 plasmid mutant library | CGAAAC |
| Bris07 post-selection mutant library (Replicate 1) | CGTACG |
| Bris07 post-selection mutant library (Replicate 2) | CCACTC |
| Bris07 post-selection mutant library (Replicate 3) | GCTACC |
| Bris07 rev6 plasmid mutant library | GGCCAC |
| Bris07 rev6 post-selection mutant library (Replicate 1) | CGAAAC |
| Bris07 rev6 post-selection mutant library (Replicate 2) | CGTACG |
| Bris07 rev6 post-selection mutant library (Replicate 3) | CCACTC |
| HK68 WT post-selection mutant library (Replicate 1) | CGTGAT |
| HK68 WT post-selection mutant library (Replicate 2) | ACATCG |
| HK68 WT post-selection mutant library (Replicate 3) | GCCTAA |
| HK68 E190D post-selection mutant library (Replicate 1) | TGGTCA |
| HK68 E190D post-selection mutant library (Replicate 2) | CACTGT |
| HK68 E190D post-selection mutant library (Replicate 3) | ATTGCC |
| Vic11 WT post-selection mutant library (Replicate 1) | GATCTG |
| Vic11 WT post-selection mutant library (Replicate 2) | TCAAGT |
| Vic11 WT post-selection mutant library (Replicate 3) | CTGATC |
| Vic11 D190E post-selection mutant library (Replicate 1) | AAGCTA |
| Vic11 D190E post-selection mutant library (Replicate 2) | GTAGCC |
| Vic11 D190E post-selection mutant library (Replicate 3) | TACAAG |
| HK68 WT plasmid mutant library | TTGACT |
| HK68 E190D plasmid mutant library | GGAACT |
| Vic11 WT plasmid mutant library | TGACAT |
| Vic11 D190E plasmid mutant library | GGACGG |

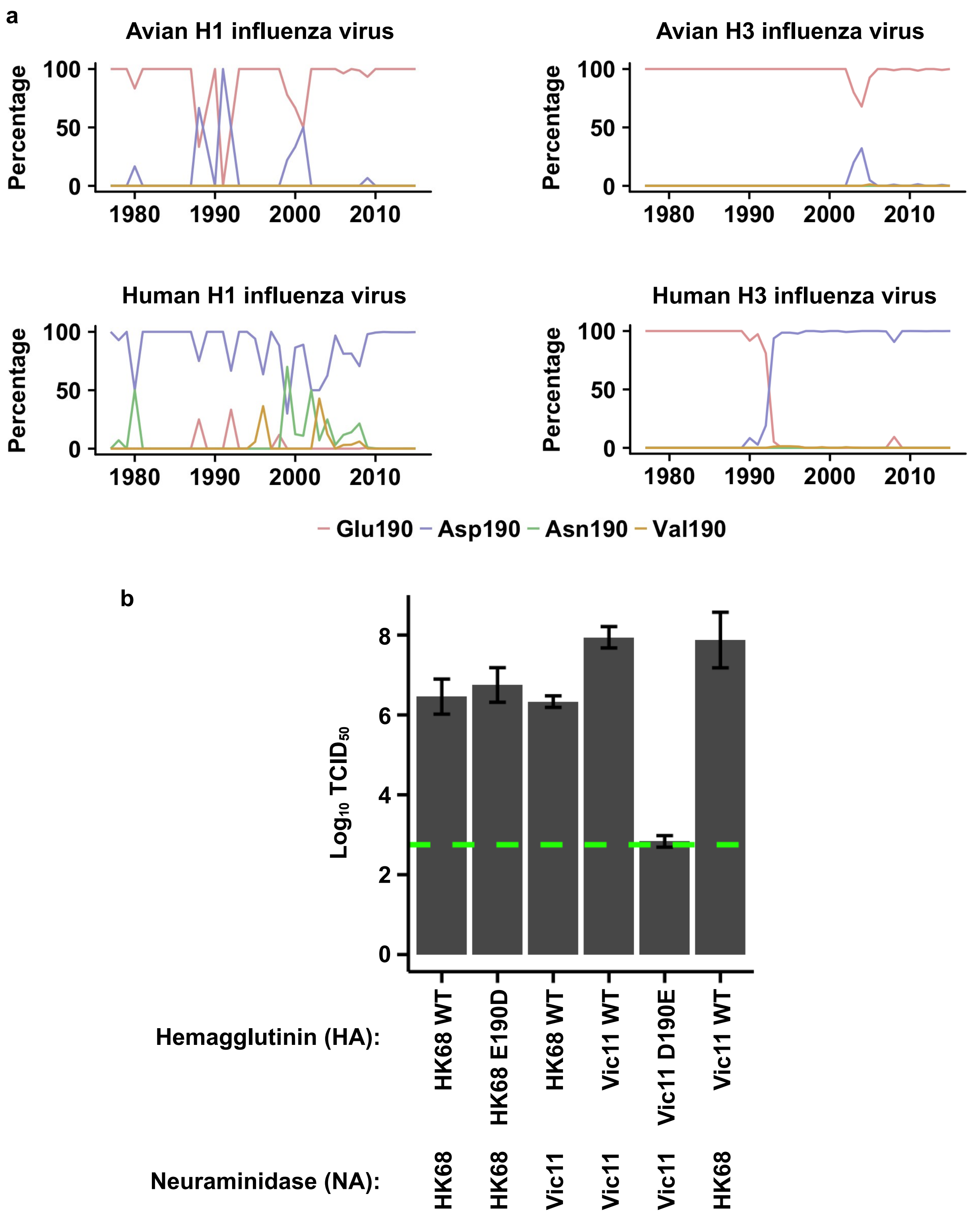


**Supplementary Figure 1. Fitness effect of exchanging the ancestor and descendant states at residue 190. (a)** Hemagglutinin (HA) protein sequences were downloaded from Global Initiative for Sharing Avian Influenza Data (GISAID; http://gisaid.org). The occurrence frequencies of different amino acids at residue 190 in different years since 1977 are shown. During the course of human H3N2 evolution, HA residue 190 changed from Glu to Asp. **(b)** The fitness effects of D190E in a recent strain A/Victoria/361/2011 (Vic11) and of E190D in the ancestor strain A/Hong Kong/1/1968 (HK68) were examined by virus rescue experiment. The effect of swapping neuraminidase (NA) was also examined, that is pairing the HA from HK68 with NA from Vic11 and vice versa. Virus titer was measured by $TCID_{50}$. Error bars indicate the standard deviation of three independent experiments. “WT” indicates wild type. The green dashed line represents the lower detection limit.

```
                         11        21        31        41        51        61        71        81
A/Hong Kong/1/1968       ATLCLGHHAVPNGTLVKTITDDQIEVTNATELVQSSSTGKICNNPHRILDGIDCTLIDALLGDPHCDVFQNETWDLFVER
A/Shangdong/9/1993       ATLCLGHHAVPNGTLVKTITNDQIEVTNATELVQSSSTGRICGSPHRILDGKNCTLIDALLGDPHCDGFQNKEWDLFVER
A/Moscow/10/1999         ATLCLGHHAVPNGTLVKTITNDQIEVTNATELVQSSSTGRICDSPHQILDGENCTLIDALLGDPHCDGFQNKEWDLFVER
A/Wyoming/3/2003         ATLCLGHHAVPNGTIVKTITNDQIEVTNATELVQSSSTGGICDSPHQILDGENCTLIDALLGDPQCDGFQNKKWDLFVER
A/Brisbane/10/2007       ATLCLGHHAVPNGTIVKTITNDQIEVTNATELVQSSSTGEICDSPHQILDGENCTLIDALLGDPQCDGFQNKKWDLFVER
A/Perth/16/2009          ATLCLGHHAVPNGTIVKTITNDQIEVTNATELVQSSSTGEICDSPHQILDGKNCTLIDALLGDPQCDGFQNKKWDLFVER
A/Victoria/361/2011      ATLCLGHHAVPNGTIVKTITNDQIEVTNATELVQNSSIGEICDSPHQILDGENCTLIDALLGDPQCDGFQNKKWDLFVER
A/Michigan/15/2014       ATLCLGHHAVPNGTIVKTITNDRIEVTNATELVQNSSIGEICDSPHQILDGENCTLIDALLGDPQCDGFQNKKWDLFVER
A/North Dakota/26/2016   ATLCLGHHAVPNGTIVKTITNDRIEVTNATELVQNSSIGEICDSPHQILDGENCTLIDALLGDPQCDGFQNKKWDLFVER
                         91        101       111       121       131       141       151       161
A/Hong Kong/1/1968       SKAFSNCYPYDVPDYASLRSLVASSGTLEFITEGFTWTGVTQNGGSNACKRGPGSGFFSRLNWLTKSGSTYPVLNVTMPN
A/Shangdong/9/1993       SKAYSNCYPYDVPDYASLRSLVASSGTLEFINEDFNWTGVAQDGGSYACKRGSVNSFFSRLNWLHKLEYKYPALNVTMPN
A/Moscow/10/1999         SKAYSNCYPYDVPDYASLRSLVASSGTLEFNNESFNWTGVAQNGTSSACKRRSINSFFSRLNWLHQLKYRYPALNVTMPN
A/Wyoming/3/2003         SKAYSNCYPYDVPDYASLRSLVASSGTLEFNNESFNWAGVTQNGTSSACKRRSNKSFFSRLNWLTHLKYKYPALNVTMPN
A/Brisbane/10/2007       SKAYSNCYPYDVPDYASLRSLVASSGTLEFNNESFNWTGVTQNGTSSACIRRSNNSFFSRLNWLTHLKFKYPALNVTMPN
A/Perth/16/2009          SKAYSNCYPYDVPDYASLRSLVASSGTLEFNNESFNWTGVTQNGTSSACIRRSKNSFFSRLNWLTHLNFKYPALNVTMPN
A/Victoria/361/2011      SKAYSNCYPYDVPDYASLRSLVASSGTLEFNNESFNWTGVTQNGTSSACIRRSNNSFFSRLNWLTHLNFKYPALNVTMPN
A/Michigan/15/2014       SKAYSNCYPYDVPDYASLRSLVASSGTLEFNNESFNWTGVTQNGTSSACIRRSSSSFFSRLNWLTHLNYKYPALNVTMPN
A/North Dakota/26/2016   SKAYSNCYPYDVPDYASLRSLVASSGTLEFNNESFNWAGVTQNGTSSSCIRGSNSSFFSRLNWLTHLNSKYPALNVTMPN
                         171       181       191       201       211       221       231       241
A/Hong Kong/1/1968       NDNFDKLYIWGVHHPSTNQEQTSLYVQASGRVTVSTRRSQQTIIPNIGSRPWVRGLSSRISIYWTIVKPGDVLVINSNGN
A/Shangdong/9/1993       NGKFDKLYIWGVHHPSTDSDQTSLYVRASGRVTVSTKRSQQTVTPNIGSRPWVRGQSSRISIYWTIVKPGDILLINSTGN
A/Moscow/10/1999         NDKFDKLYIWGVHHPSTDSDQTSLYTQASGRVTVSTKRSQQTVIPNIGSRPWVRGISSRISIYWTIVKPGDILLIKSTGN
A/Wyoming/3/2003         NEKFDKLYIWGVHHPVTDSDQISLYAQASGRITVSTKRSQQTVIPNIGFRPRVRDISSRISIYWTIVKPGDILLINSTGN
A/Brisbane/10/2007       NEKFDKLYIWGVHHPGTDNDQIFPYAQASGRITVSTKRSQQTVIPNIGSRPRVRNIPSRISIYWTIVKPGDILLINSTGN
A/Perth/16/2009          NEQFDKLYIWGVHHPGTDKDQIFLYAQASGRITVSTKRSQQTVSPNIGSRPRVRNIPSRISIYWTIVKPGDILLINSTGN
A/Victoria/361/2011      NEQFDKLYIWGVHHPGTDKDQIFLYAQSSGRITVSTKRSQQAVIPNIGSRPRIRNIPSRISIYWTIVKPGDILLINSTGN
A/Michigan/15/2014       NEQFDKLYIWGVHHPGTDKDQIFLYAQSSGRITVSTKRSQQAVIPNIGSRPKIRDIPSRISIYWTIVKPGDILLINSTGN
A/North Dakota/26/2016   NEQFDKLYIWGVHHPGTDKDQIFLYAQPSGRITVSTKRSQQAVIPNIGSRPRIRDIPSRISIYWTIVKPGDILLINSTGN
                         251       261       271       281       291       301       311       321
A/Hong Kong/1/1968       LIAPRGYFKMRTGKSSIMRSDAPIDTCISECITPNGSIPNDKPFQNVNKITYGACPKYVKQNTLKLATGMRNVPEKQTR
A/Shangdong/9/1993       LIAPRGYFKIRNGKSSIMRSDAPIGNCSSECITPNGSIPNDKPFQNVNRITYGACPRYVKQNTLKLATGMRNVPEKQTR
A/Moscow/10/1999         LIAPRGYFKIRSGKSSIMRSDAPIGKCNSECITPNGSIPNDKPFQNVNRITYGACPRYVKQNTLKLATGMRNVPEKQTR
A/Wyoming/3/2003         LIAPRGYFKIRSGKSSIMRSDAPIGKCNSECITPNGSIPNDKPFQNVNRITYGACPRYVKQNTLKLATGMRNVPEKQTR
A/Brisbane/10/2007       LIAPRGYFKIRSGKSSIMRSDAPIGKCNSECITPNGSIPNDKPFQNVNRITYGACPRYVKQNTLKLATGMRNVPEKQTR
A/Perth/16/2009          LIAPRGYFKIRSGKSSIMRSDAPIGKCNSECITPNGSIPNDKPFQNVNRITYGACPRYVKQNTLKLATGMRNVPEKQTR
A/Victoria/361/2011      LIAPRGYFKIRSGKSSIMRSDAPIGKCNSECITPNGSIPNDKPFQNVNRITYGACPRYVKQSTLKLATGMRNVPEKQTR
A/Michigan/15/2014       LIAPRGYFKIRSGKSSIMRSDAPIGKCKSECITPNGSIPNDKPFQNVNRITYGACPRYVKHSTLKLATGMRNVPEKQTR
A/North Dakota/26/2016   LIAPRGYFKIRSGKSSIMRSDAPIGKCKSECITPNGSIPNDKPFQNVNRITYGACPRYVKQNTLKLATGMRNVPERQTR
```

**Supplementary Figure 2. Multiple sequence alignment of HA1.** The HA1 sequences for strains being used in this study, namely A/Shangdong/9/1993 (Shang93), A/Moscow/10/1999 (Mos99), A/Wyoming/3/2003 (Wy03), A/Brisbane/10/2007 (Bris07), A/Perth/16/2009 (Perth09), Vic11, A/Michigan/15/2014 (Mich14), and A/North Dakota/26/2016 (NDako16). Residues that are not completely conserved among these sequences are highlighted in pink. Alignment was performed using (http://www.bioinformatics.org/sms/multi_align.html)[1].

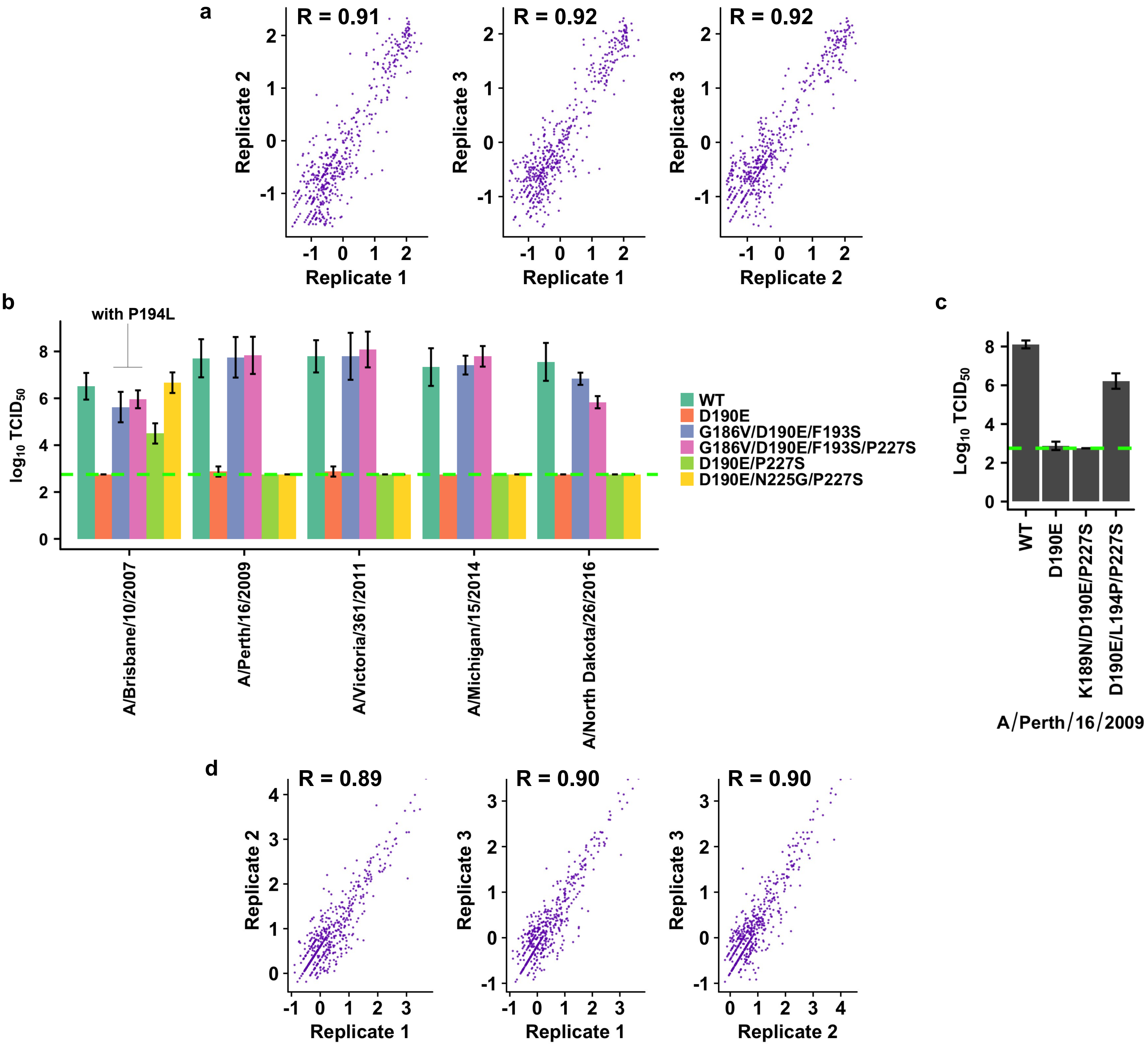


**Supplementary Figure 3. Systematic mapping of mutations that restored E190D reversibility. (a)** A deep mutational scanning experiment was performed in triplicate to investigate the fitness effect for all genotypes across nine biallelic sites, namely residues 145 (Asn or Lys), 186 (Gly or Val), 189 (Asn or Ser), 190 (Asp or Glu), 193 (Phe or Ser), 194 (Leu or Pro), 219 (Ser and Phe), 225 (Asn and Asp), and 227 (Pro and Ser) on Bris07 hemagglutinin. Correlations of the $log_{10}$ RF index for individual variants between each indicated replicate are shown as scatterplots. Each data point within a scatterplot represents a unique variant. The Pearson correlation between each replicate is indicated. **(b)** Effects of different mutations on replication fitness of Perth09, Vic11, Mich14, and NDako16 were examined by virus rescue experiment. These mutations were able to compensate the lethality of D190E in Bris07. The data for Bris07 are the same as that in Fig. 2c, and are shown here for comparison purpose. Virus titer was measured by $TCID_{50.}$ Error bars indicate the standard deviation of three independent experiments. The green dashed line represents the lower detection limit. **(c)** Effects of different mutations on replication fitness Perth09 were examined by virus rescue experiment. Virus titer was measured by $TCID_{50.}$ Error bars indicate the standard deviation of three independent experiments. The green dashed line represents the lower detection limit. **(d)** A deep mutational scanning experiment was performed in triplicate to investigate the fitness effect for all genotypes across nine biallelic sites, namely residues 155 (Thr or His), 156 (His or Gln), 159 (Phe or Tyr), 160 (Lys or Arg), 189 (Asn or Ser), 192 (Ile or Thr), 196 (Ala and Thr), 202 (Ile and Val), and 222 (Arg and Trp) on Bris07 hemagglutinin in a genetic background of G186S/D190E/F193S/P194L/N225G/P227S (rev6). Correlations of the $log_{10}$ RF index for individual variants between each indicated replicate are shown as scatterplots. Each data point within a scatterplot represents a unique variant. The Pearson correlation between each replicate is indicated.

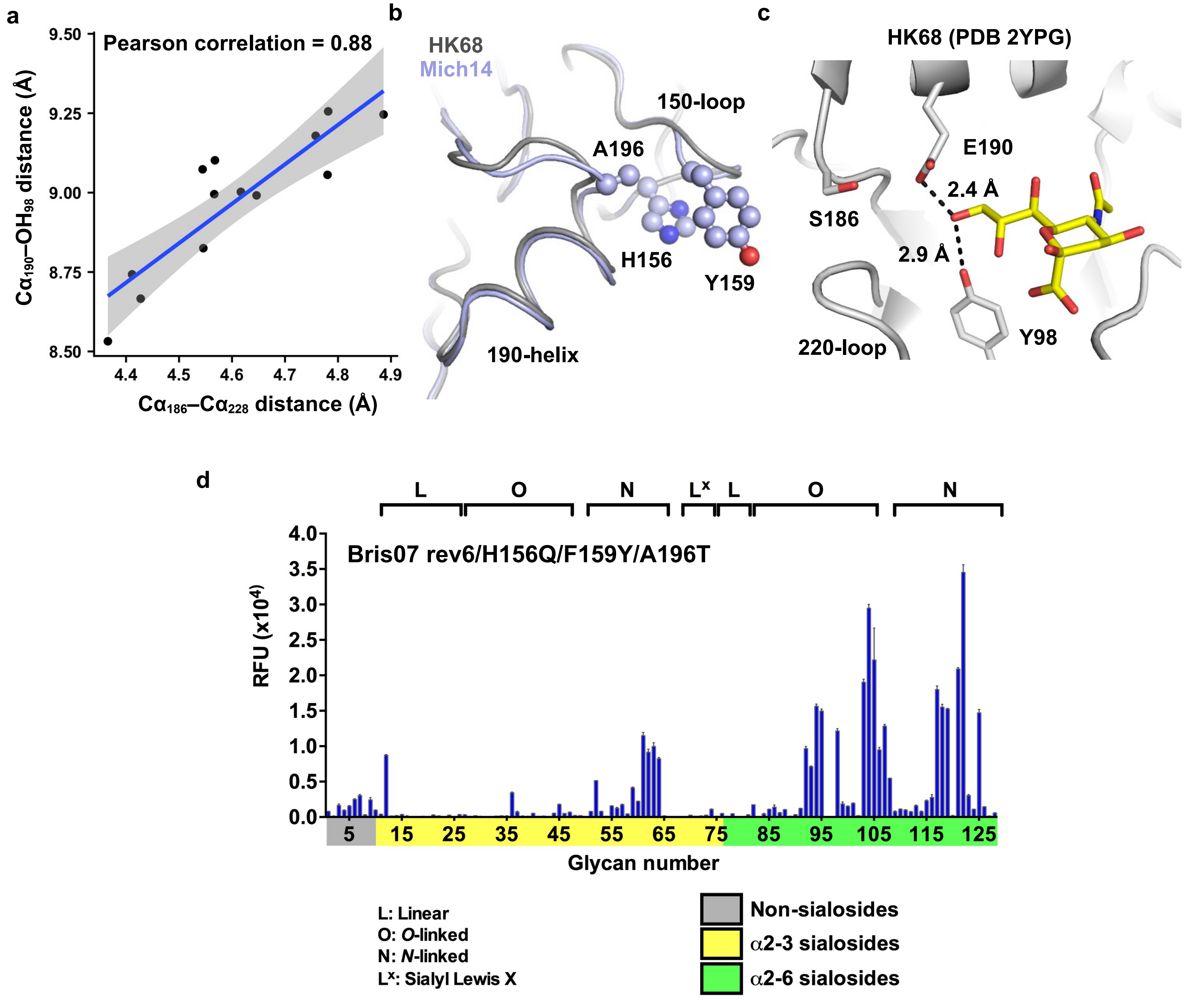


**Supplementary Figure 4. Structural analysis of HA RBS of human H3N2 strains. (a)** The relationship between the $C\alpha_{186}$–$C\alpha_{228}$ distance and the $C\alpha_{190}$–$OH_{98}$ distance is shown as a scatterplot. The data are the same as that in Figure 4B. **(b)** The locations of residues 156, 159, and 196 (sphere representation) on Mich14 (blue) are shown. For comparison, HK68 (grey) is aligned to Mich14. HK68: PDB 4FNK[2]. **(c)** The interaction between the RBS of HK68 and sialic acid (yellow sticks) is shown. PDB 2YPG[3]. **(d)** 293S-expressed recombinant Bris07 rev6/H156Q/F159Y/A196T HA was purified and analyzed by sialoside glycan array.

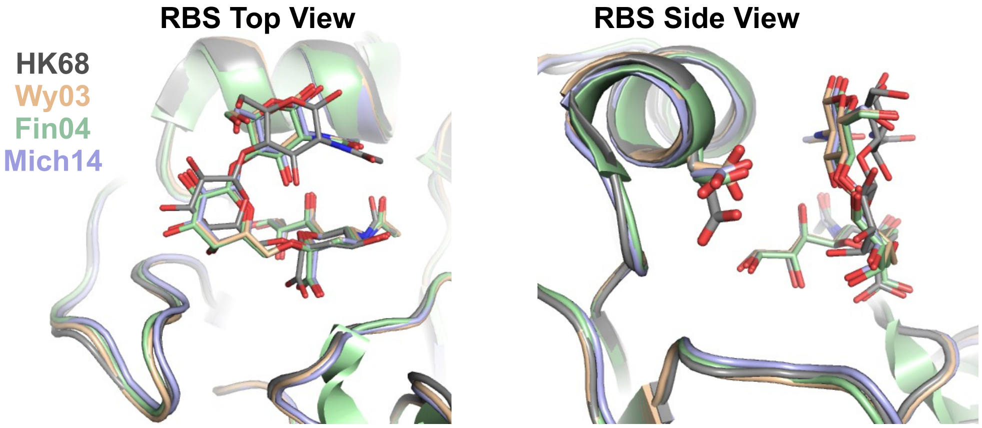


**Supplementary Figure 5. Comparison of receptor binding of human H3N2 strains.** The orientations of human receptor analog 6'-SLN in complex with HK68 (PDB 2YPG[3]), Wy03, Fin04 (PDB 2YP4[3]), and Mich14 are compared.

HK68 E190D

Wy03 WT

Wy03 D190E

Mich14 WT

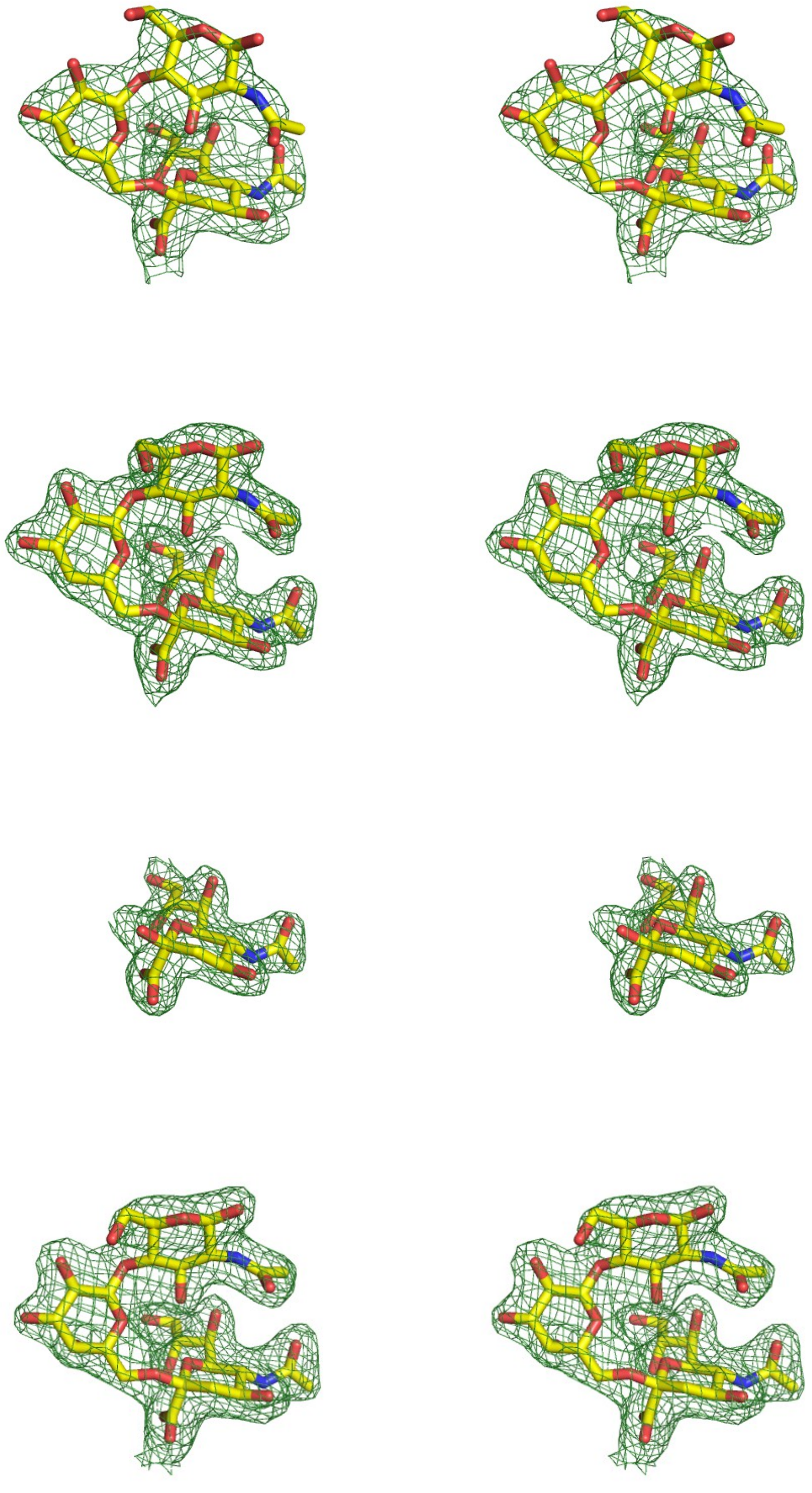

**Supplementary Figure 6. Final 2Fo-Fc electron density maps for glycan receptors (6'-SLN).** Stereo representation of the final 2Fo-Fc electron density maps for the glycan receptors (6'-SLN) is contoured at 0.8 σ.

HK68 E190D

Wy03 WT

Wy03 D190E

Mich14 WT

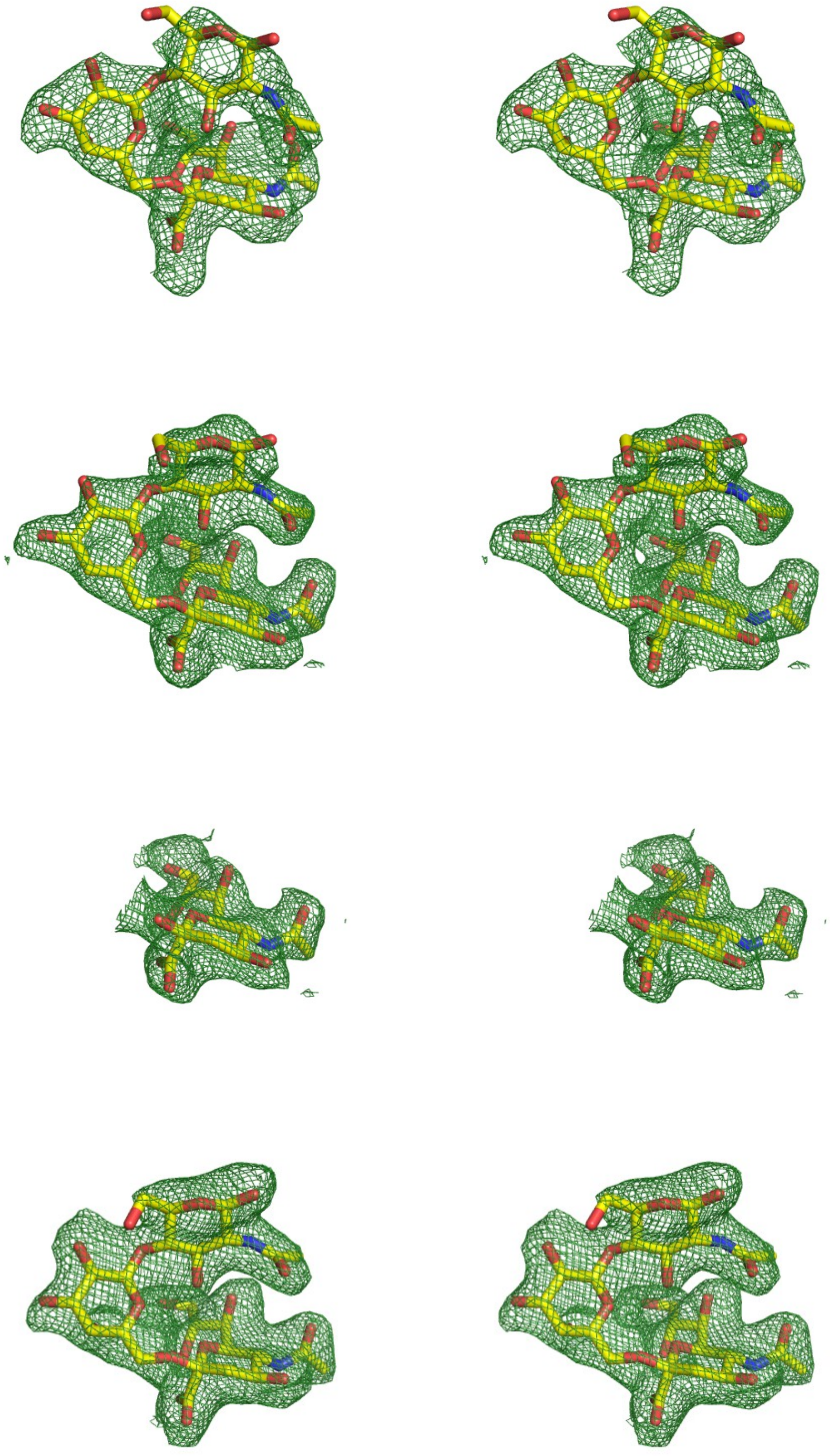

**Supplementary Figure 7. Omit (Fo-Fc) electron density maps for glycan receptors (6'-SLN).** Stereo representation of the omit (Fo-Fc) electron density maps for the glycan receptors (6'-SLN) is contoured at 2.0 σ.

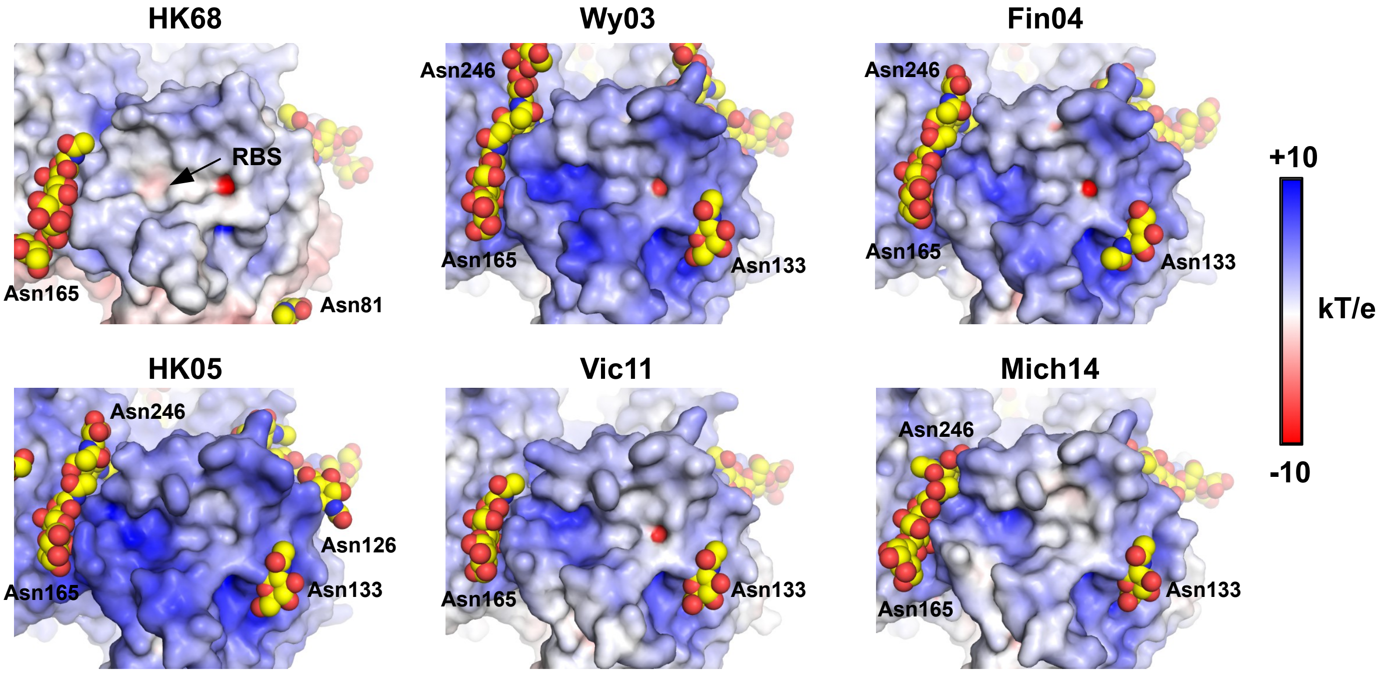


**Supplementary Figure 8. Evolution of HA RBS of human H3N2 strains.** Surface electrostatics representation of the RBS of HK68, Wy03, Fin04, HK05, Vic11, and Mich14. HK68: PDB 4FNK[2]. Fin04: PDB 2YP2[3]. HK05: PDB 2YP7[3]. Vic11: PDB 4O5N[4]. Glycans are shown in sphere representation and colored by atom types (yellow for carbon, red for oxygen, and blue for nitrogen).

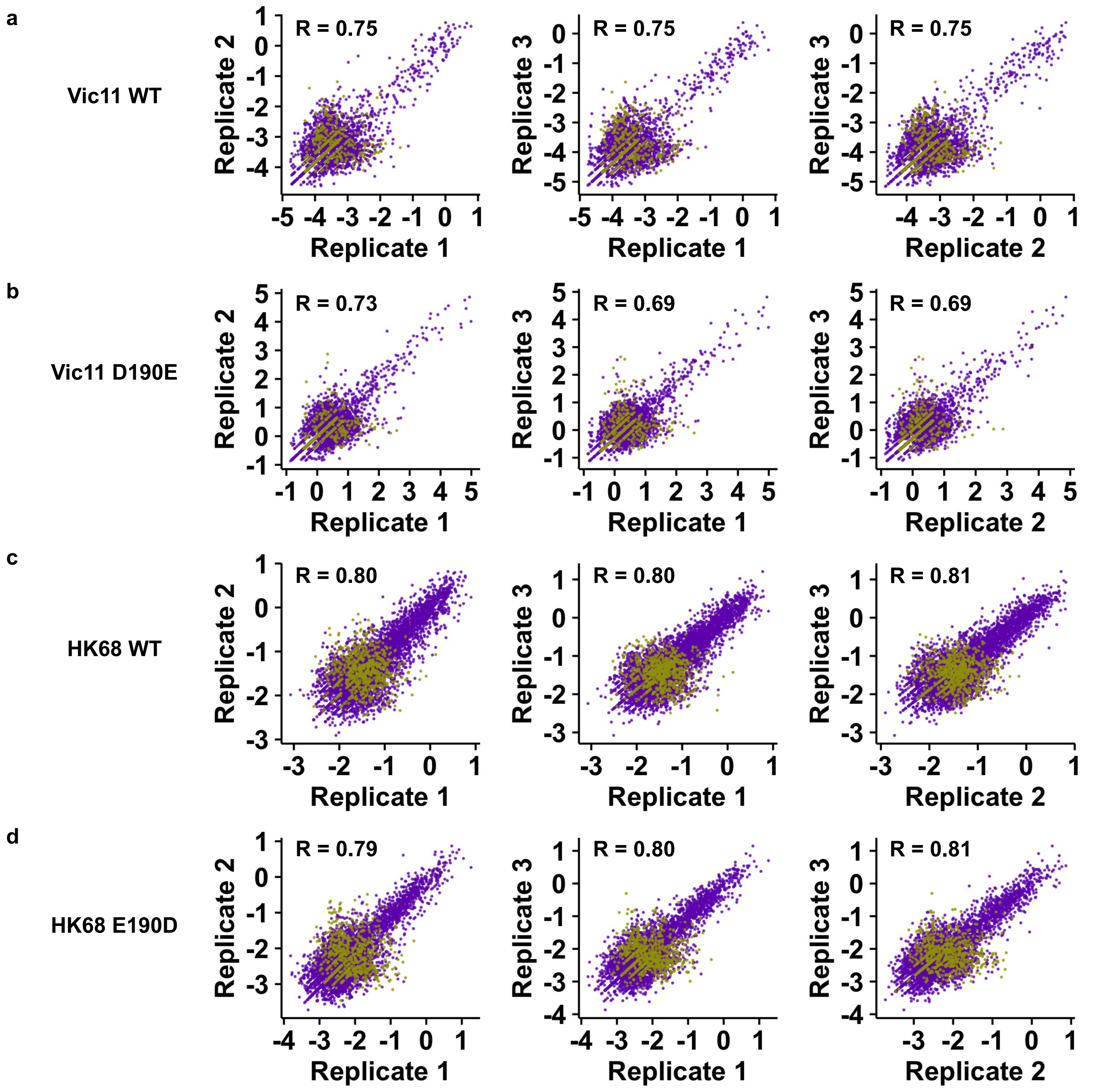


**Supplementary Figure 9. Correlation between replicates of deep mutational scanning across residues 225, 226 and 228 in Vic11 and HK68 HAs.** A total of four deep mutational scanning experiments were performed in triplicate for HA residues 225, 226 and 228 in Vic11 under WT or D190E genetic backgrounds, and in HK68 under WT or E190D genetic backgrounds. Correlations of the $\log_{10}$ RF index for individual variants between each indicated replicate for **(a)** Vic11 WT genetic background, **(b)** Vic11 D190E genetic background, **(c)** HK68 WT genetic background, and **(d)** HK68 E190D genetic backgrounds are shown as scatterplots. Each data point within a scatterplot represents a unique variant. Missense variants are colored in purple. Nonsense variants are colored in greenish-yellow. The Pearson correlation between each replicate is indicated.

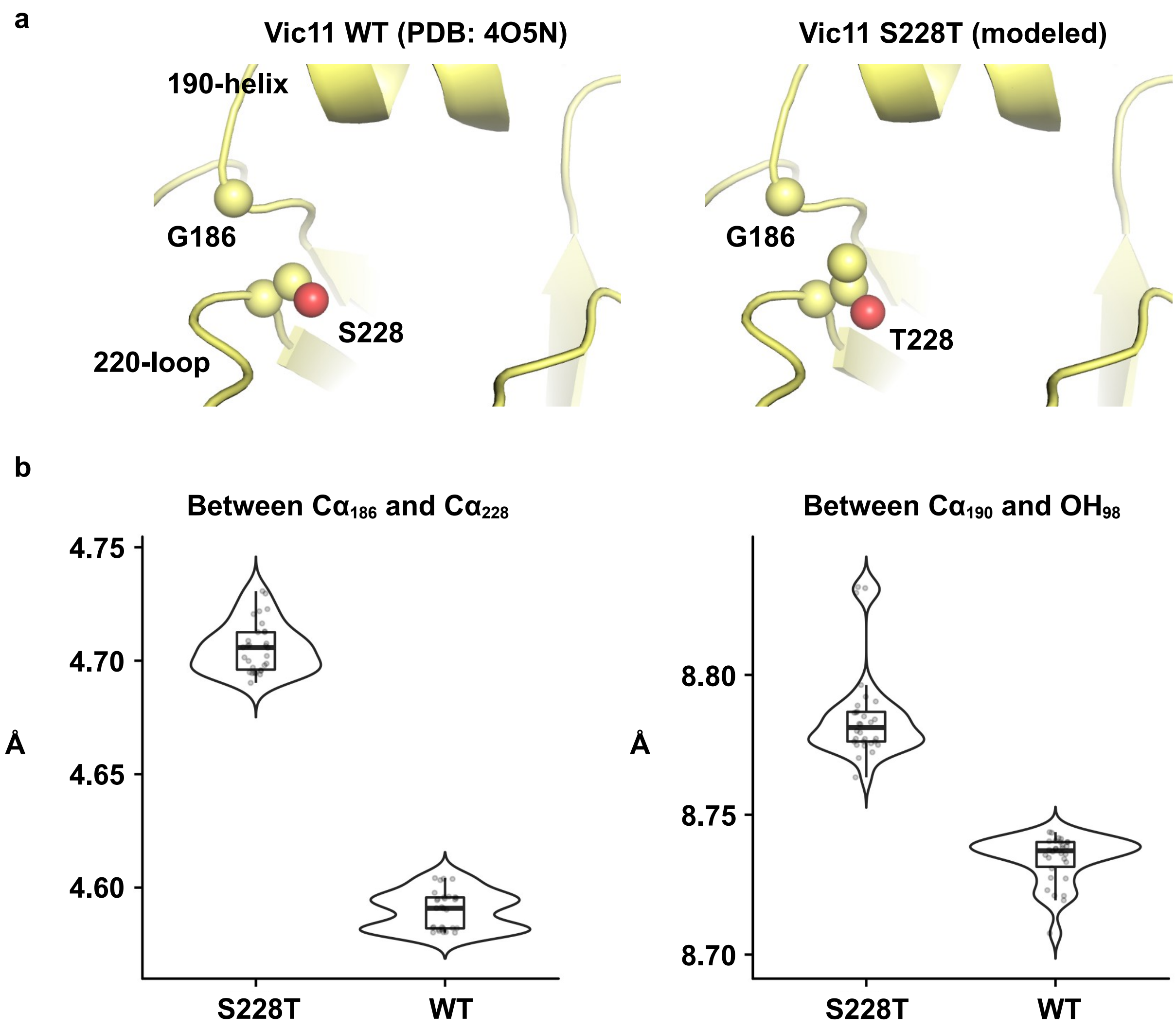


**Supplementary Figure 10. Possible role of S228T in adjusting the height of the 190-helix in Vic11. (a)** Structure of Vic11 S228T was modeled by the ddg_monomer application in Rosetta software[5] based on PDB 4O5N[4]. Parameters from row 16 of Table I in Kellogg et al. 2011[6] were used. 30 independent energy minimization runs were performed on each of Vic11 WT and S228T. The representative structures of the energy minimized models of Vic11 WT and S228T are shown. **(b)** The distances between the Cα of residues 186 ($C\alpha_{186}$) and the Cα of residues 228 ($C\alpha_{228}$) and between the Cα of residues 190 ($C\alpha_{190}$) and the phenolic oxygen of residue 98 ($OH_{98}$) were measured. Each datapoint (grey circle) represent the result from each of the 30 energy minimization runs. The distributions are shown as a box plot overlaid with a violin plot. The $C\alpha_{186}$-$C\alpha_{228}$ distance in Vic11 S228T is significantly larger than that in Vic11 WT ($P < 2.2e-16$, two tailed t-test). The $C\alpha_{190}$-$OH_{98}$ distance in Vic11 S228T is also significantly larger than that in Vic11 WT ($P < 2.2e-16$, two tailed t-test).

| A/Brisbane/10/2007 | | HAI titer | | | | HAI concentration (μg/ml) | | | |
|---|---|---|---|---|---|---|---|---|---|
| | | Virus only | Normal sera | Anti-sera Bris/07 (H3N2) | Anti-sera R-Bris/07 HA | mAb R-Bris/07 HA (FR509) | mAb R-Bris/07 HA (FR510) | mAb R-Bris/07 HA (FR511) | mAb R-Bris/07 HA (FR512) |
| | WT | 0 | 20 | 2560 | **5120** | 1.25 | >10.0 | 10.0 | 5.0 |
| | G186V/D190E/F193S/P194L | 0 | 20 | 0 | 320 | **0.625** | >10.0 | 5.0 | 2.5 |
| | G186V/D190E/F193S/P194L/P227S | 0 | 40 | 160 | 320 | **0.625** | 10.0 | 2.5 | 2.5 |
| | D190E/N225G/P227S | 0 | 40 | 2560 | **5120** | 0.938 | >10.0 | 7.5 | 2.5 |
| | rev6/A196T/H156Q/F159Y | 0 | 40 | 160 | 320 | **0.625** | >10.0 | 2.5 | 2.5 |

**Supplementary Figure 11. Hemagglutination inhibition (HAI) assay.** The change of antigenicity in G186V/D190E/F193S/P194L, G186V/D190E/F193S/P194L/P227S, D190E/N225G/P227S, and rev6/H156Q/F159Y/A196T was tested using three serum samples and four monoclonal antibodies.

## Supplementary Figure 12. Glycan array compound list

| Glycan # | Common Name | Structure |
|---|---|---|
| 1 | Galβ(1-4)GlcNAcβ-ethyl-NH2 | 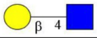 |
| 2 | Galβ(1-4)GlcNAcβ(1-3)Galβ(1-3)GalNAcα-Thr-NH2 | 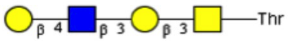  |
| 3 | Galβ(1-4)GlcNAcβ(1-6)[Galβ(1-3)]-GalNAcα-Thr-NH2 | 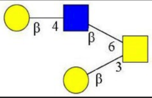 |
| 4 | Galβ(1-4)GlcNAcβ(1-3)GalNAcα-Thr-NH2 | 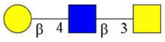 |
| 5 | Galβ(1-4)GlcNAcβ(1-3)[Galβ(1-4)GlcNAcβ(1-6)]-GalNAcα-Thr-NH2 | 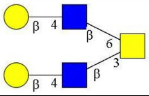 |
| 6 | Galβ(1-4)GlcNAcβ(1-6)GalNAcα-Thr-NH2 | 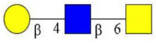 |
| 7 | Galβ(1-4)GlcNAcβ(1-2)Manα (1-3)[Galβ(1-4)GlcNAcβ(1-2)Manα(1-6)]-Manβ(1-4)GlcNAcβ(1-4)GlcNAcβ-Asn-NH2 | 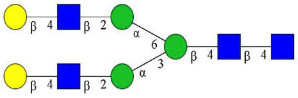 |
| 8 | Galβ(1-4)GlcNAcβ(1-2)Manα(1-3)[Galβ(1-4)GlcNAcβ(1-2)Manα(1-6)]-Manβ(1-4)GlcNAcβ(1-4)[Fucα(1-6)]-GlcNAcβ-Asn-Ser-Thr-NH2 | 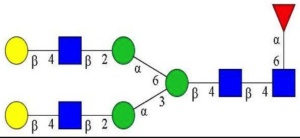 |
| 9 | Galβ(1-4)GlcNAcβ(1-2)Manα(1-3){Galβ(1-4)GlcNAcβ(1-2)[Galβ(1-4)GlcNAcβ(1-2)]-Manα(1-6)}-Manβ(1-4)GlcNAcβ(1-4)GlcNAcβ-Asn-Lys-NH2 | 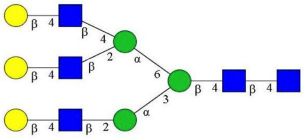 |
| 10 | Galβ(1-4)GlcNAcβ(1-2)Manα(1-3){Galβ(1-4)GlcNAcβ(1-2)[Galβ(1-4)GlcNAcβ(1-2)]-Manα(1-6)}-Manβ(1-4)GlcNAcβ(1-4)[Fucα(1-6)]-GlcNAcβ-(Lys-Val-Ala)Asn-Lys-Thr-NH2 | 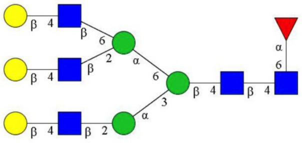 |
| 11 | NeuAcα(2-3)Galβ(1-4)6-O-sulfo-GlcNAcβ-propyl-NH2 | 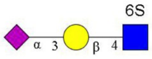  |
| 12 | NeuAcα(2-3)Galβ(1-4)[Fucα(1-3)]-6-O-sulfo-GlcNAcβ-propyl-NH2 | 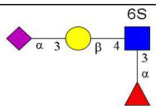  |
| 13 | NeuAcα(2-3)6-O-sulfo-Galβ(1-4)GlcNAcβ-ethyl-NH2 | 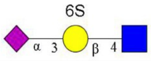  |
| 14 | NeuAcα(2-3)6-O-sulfo-Galβ(1-4)[Fucα(1-3)]-GlcNAcβ-propyl-NH2 | 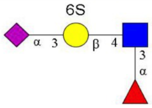  |
| 15 | NeuAcα(2-3)Galβ(1-3)6-O-sulfo-GlcNAcβ-propyl-NH2 | 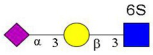  |
| 16 | NeuAcα(2-3)Galβ(1-4)Glcβ-ethyl-NH2 | 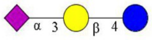 |
| 17 | NeuAcα(2-3)Galβ(1-4)GlcNAcβ-ethyl-NH2 | 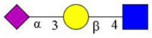 |
| 18 | NeuAcα(2-3)Galβ(1-4)GlcNAcβ(1-3)Galβ(1-4)GlcNAcβ-ethyl-NH2 | 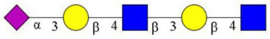 |
| 19 | NeuAcα(2-3)Galβ(1-4)GlcNAcβ(1-3)Galβ(1-4)GlcNAcβ(1-3)Galβ(1-4)GlcNAcβ-ethyl-NH2 | 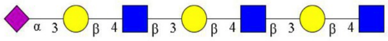 |

| Glycan # | Common Name | Structure |
|---|---|---|
| 20 | NeuAcα(2-3)GalNAcβ(1-4)GlcNAcβ-ethyl-NH2 | 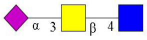 |
| 21 | NeuAcα(2-3)Galβ(1-3)GlcNAcβ-ethyl-NH2 | 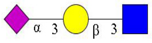 |
| 22 | NeuAcα(2-3)Galβ(1-3)GlcNAcβ(1-3)Galβ(1-4)GlcNAcβ-ethyl-NH2 | 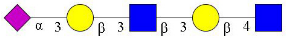 |
| 23 | NeuAcα(2-3)Galβ(1-3)GlcNAcβ(1-3)Galβ(1-3)GlcNAcβ-ethyl-NH2 | 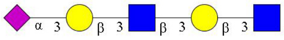 |
| 24 | NeuAcα(2-3)Galβ(1-3)GalNAcβ(1-3)Galα(1-4)Galβ(1-4)Glcβ-ethyl-NH2 | 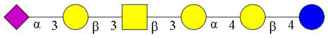 |
| 25 | NeuAcα(2-3)Galβ(1-3)GalNAcα-Thr-NH2 | 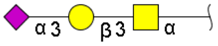 |
| 26 | NeuAcα(2-3)Galβ(1-4)GlcNAcβ(1-3)Galβ(1-3)GalNAcα-Thr-NH2 | 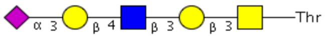 |
| 27 | NeuAcα(2-3)Galβ(1-4)GlcNAcβ(1-3)Galβ(1-4)GlcNAcβ(1-3)Galβ(1-3)GalNAcα-Thr-NH2 | 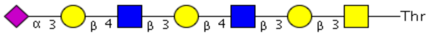 |
| 28 | NeuAcα(2-3)Galβ(1-4)GlcNAcβ(1-3)Galβ(1-4)GlcNAcβ(1-3)Galβ(1-4)GlcNAcβ(1-3)Galβ(1-3)GalNAcα-Thr-NH2 | 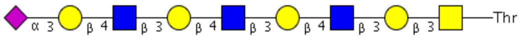 |
| 29 | NeuAcα(2-3)Galβ(1-4)GlcNAcβ(1-3)Galβ(1-4)GlcNAcβ(1-3)Galβ(1-4)GlcNAcβ(1-3)Galβ(1-4)GlcNAcβ(1-3)Galβ(1-3)GalNAcα-Thr-NH2 | 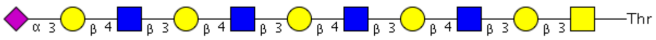 |
| 30 | NeuAcα(2-3)Galβ(1-4)GlcNAcβ(1-3)Galβ(1-4)GlcNAcβ(1-3)Galβ(1-4)GlcNAcβ(1-3)Galβ(1-4)GlcNAcβ(1-3)Galβ(1-4)GlcNAcβ(1-3)Galβ(1-3)GalNAcα-Thr-NH2 | 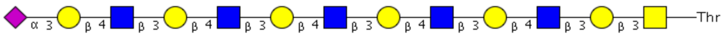 |
| 31 | NeuAcα(2-3)Galβ(1-4)GlcNAcβ(1-6)[Galβ(1-3)]-GalNAcα-Thr-NH2 | 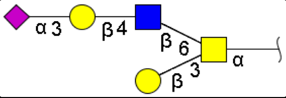 |
| 32 | NeuAcα(2-3)Galβ(1-4)GlcNAcβ(1-3)Galβ(1-4)GlcNAcβ(1-6)[Galβ(1-3)]-GalNAcα-Thr-NH2 | 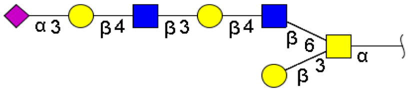 |
| 33 | NeuAcα(2-3)Galβ(1-4)GlcNAcβ(1-3)Galβ(1-4)GlcNAcβ(1-3)Galβ(1-4)GlcNAcβ(1-6)[Galβ(1-3)]-GalNAcα-Thr-NH2 | 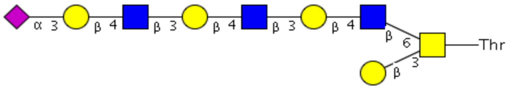 |
| 34 | NeuAcα(2-3)Galβ(1-4)GlcNAcβ(1-3)Galβ(1-4)GlcNAcβ(1-3)Galβ(1-4)GlcNAcβ(1-3)Galβ(1-4)GlcNAcβ(1-6)[Galβ(1-3)]-GalNAcα-Thr-NH2 | 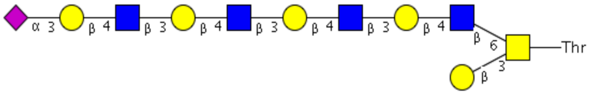 |
| 35 | NeuAcα(2-3)Galβ(1-4)GlcNAcβ(1-3)Galβ(1-4)GlcNAcβ(1-3)Galβ(1-4)GlcNAcβ(1-3)Galβ(1-4)GlcNAcβ(1-3)Galβ(1-4)GlcNAcβ(1-6)[Galβ(1-3)]-GalNAcα-Thr-NH2 | 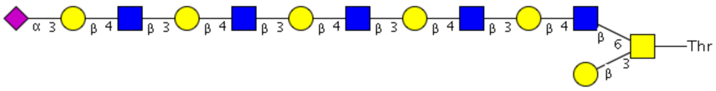 |
| 36 | NeuAcα(2-3)Galβ(1-4)GlcNAcβ(1-3)Galβ(1-4)GlcNAcβ(1-3)Galβ(1-4)GlcNAcβ(1-3)Galβ(1-4)GlcNAcβ(1-6)[NeuAcα(2-3)Galβ(1-4)GlcNAcβ(1-3)Galβ(1-4)GlcNAcβ(1-3)Galβ(1-4)GlcNAcβ(1-3)Galβ(1-3)]-GalNAcα-Thr-NH2 | 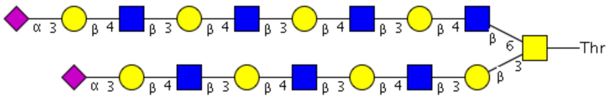 |

| Glycan # | Common Name | Structure |
|---|---|---|
| 37 | NeuAcα(2-3)Galβ(1-4)GlcNAcβ(1-3)Galβ(1-4)GlcNAcβ(1-3)Galβ(1-4)GlcNAcβ(1-3)Galβ(1-4)GlcNAcβ(1-3)Galβ(1-4)GlcNAcβ(1-6)[NeuAcα(2-3)Galβ(1-4)GlcNAcβ(1-3)Galβ(1-4)GlcNAcβ(1-3)Galβ(1-4)GlcNAcβ(1-3)Galβ(1-4)GlcNAcβ(1-3)Galβ(1-3)]-GalNAcα-Thr-NH2 | 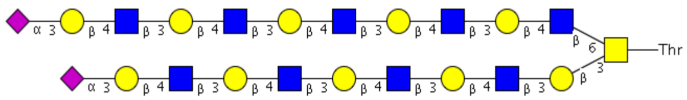 |
| 38 | NeuAcα(2-3)Galβ(1-4)GlcNAcβ(1-3)GalNAcα-Thr-NH2 | 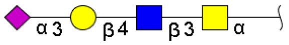 |
| 39 | NeuAcα(2-3)Galβ(1-4)GlcNAcβ(1-3)Galβ(1-4)GlcNAcβ(1-3)GalNAcα-Thr-NH2 | 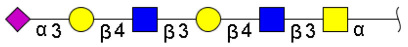 |
| 40 | NeuAcα(2-3)Galβ(1-4)GlcNAcβ(1-3)Galβ(1-4)GlcNAcβ(1-3)Galβ(1-4)GlcNAcβ(1-3)GalNAcα-Thr-NH2 | 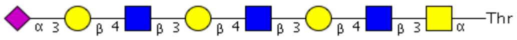 |
| 41 | NeuAcα(2-3)Galβ(1-4)GlcNAcβ(1-3)Galβ(1-4)GlcNAcβ(1-3)Galβ(1-4)GlcNAcβ(1-3)Galβ(1-4)GlcNAcβ(1-3)GalNAcα-Thr-NH2 | 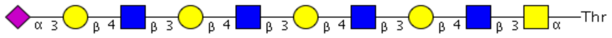 |
| 42 | NeuAcα(2-3)Galβ(1-4)GlcNAcβ(1-3)Galβ(1-4)GlcNAcβ(1-3)Galβ(1-4)GlcNAcβ(1-3)Galβ(1-4)GlcNAcβ(1-3)Galβ(1-4)GlcNAcβ(1-3)GalNAcα-Thr-NH2 | 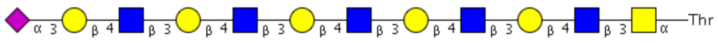 |
| 43 | NeuAcα(2-3)Galβ(1-4)GlcNAcβ(1-3)[NeuAcα(2-3)Galβ(1-4)GlcNAcβ(1-6)]-GalNAcα-Thr-NH2 | 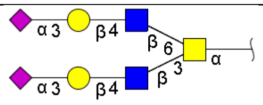 |
| 44 | NeuAcα(2-3)Galβ(1-4)GlcNAcβ(1-3)Galβ(1-4)GlcNAcβ(1-3)[NeuAcα(2-3)Galβ(1-4)GlcNAcβ(1-3)Galβ(1-4)GlcNAcβ(1-6)]-GalNAcα-Thr-NH2 | 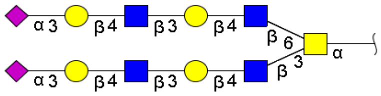 |
| 45 | NeuAcα(2-3)Galβ(1-4)GlcNAcβ(1-3)Galβ(1-4)GlcNAcβ(1-3)Galβ(1-4)GlcNAcβ(1-3)[NeuAcα(2-3)Galβ(1-4)GlcNAcβ(1-3)Galβ(1-4)GlcNAcβ(1-3)Galβ(1-4)GlcNAcβ(1-6)]-GalNAcα-Thr-NH2 | 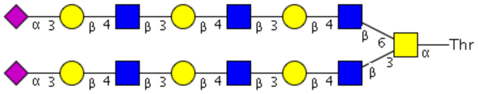 |
| 46 | NeuAcα(2-3)Galβ(1-4)GlcNAcβ(1-3)Galβ(1-4)GlcNAcβ(1-3)Galβ(1-4)GlcNAcβ(1-3)Galβ(1-4)GlcNAcβ(1-3)[NeuAcα(2-3)Galβ(1-4)GlcNAcβ(1-3)Galβ(1-4)GlcNAcβ(1-3)Galβ(1-4)GlcNAcβ(1-3)Galβ(1-4)GlcNAcβ(1-6)]-GalNAcα-Thr-NH2 | 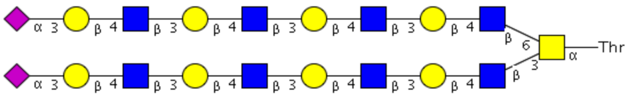 |
| 47 | NeuAcα(2-3)Galβ(1-4)GlcNAcβ(1-3)Galβ(1-4)GlcNAcβ(1-3)Galβ(1-4)GlcNAcβ(1-3)Galβ(1-4)GlcNAcβ(1-3)Galβ(1-4)GlcNAcβ(1-3)[NeuAcα(2-3)Galβ(1-4)GlcNAcβ(1-3)Galβ(1-4)GlcNAcβ(1-3)Galβ(1-4)GlcNAcβ(1-3)Galβ(1-4)GlcNAcβ(1-3)Galβ(1-4)GlcNAcβ(1-6)]-GalNAcα-Thr-NH2 | 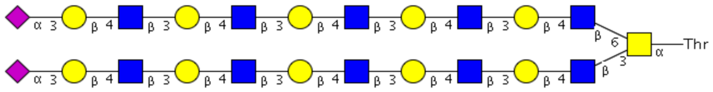 |
| 48 | NeuAcα(2-3)Galβ(1-4)GlcNAcβ(1-3)Galβ(1-4)GlcNAcβ(1-3)Galβ(1-4)GlcNAcβ(1-3)Galβ(1-4)GlcNAcβ(1-6)GalNAcα-Thr-NH2 | 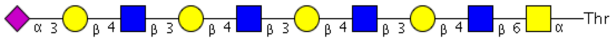 |

| Glycan # | Common Name | Structure |
|---|---|---|
| **49** | NeuAcα(2-3)Galβ(1-4)GlcNAcβ(1-3)Galβ(1-4)GlcNAcβ(1-3)Galβ(1-4)GlcNAcβ(1-3)Galβ(1-4)GlcNAcβ(1-3)Galβ(1-4)GlcNAcβ(1-6)GalNAcα-Thr-NH2 | 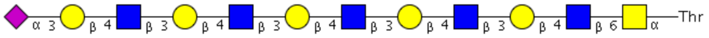 |
| **50** | NeuAcα(2-3)Galβ(1-3)GlcNAcβ(1-3)Galβ(1-4)GlcNAcβ(1-6)[NeuAcα(2-3)Galβ(1-3)GlcNAcβ(1-3)] Galβ(1-4)GlcNAcβ-ethyl-NH2 | 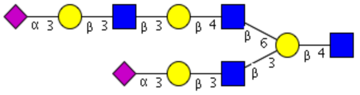 |
| **51** | NeuAcα(2-3)Galβ(1-4)GlcNAcβ(1-3)Galβ(1-4)GlcNAcβ(1-3)Galβ(1-4)GlcNAcβ(1-6)[NeuAcα(2-3)Galβ(1-4)GlcNAcβ(1-3)Galβ(1-4)GlcNAcβ(1-3)] Galβ(1-4)GlcNAcβ-ethyl-NH2 | 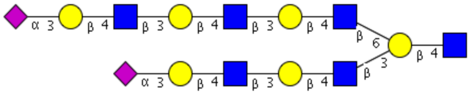 |
| **52** | NeuAcα(2-3)Galβ(1-4)GlcNAcβ(1-2)Manα(1-3)[NeuAcα(2-3)Galβ(1-4)GlcNAcβ(1-2)Manα(1-6)]-Manβ(1-4)GlcNAcβ(1-4)GlcNAcβ-Asn-NH2 | 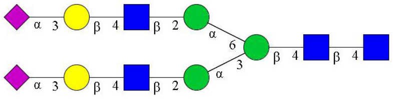 |
| **53** | NeuAcα(2-3)Galβ(1-4)GlcNAcβ(1-3)Galβ(1-4)GlcNAcβ(1-2)Manα(1-3)[NeuAcα(2-3)Galβ(1-4)GlcNAcβ(1-3)Galβ(1-4)GlcNAcβ(1-2)Manα(1-6)]-Manβ(1-4)GlcNAcβ(1-4)GlcNAcβ-Asn-NH2 | 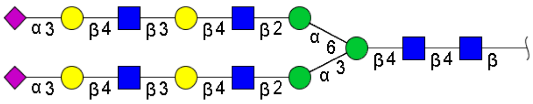 |
| **54** | NeuAcα(2-3)Galβ(1-4)GlcNAcβ(1-3)Galβ(1-4)GlcNAcβ(1-3)Galβ(1-4)GlcNAcβ(1-2)Manα(1-3)[NeuAcα(2-3)Galβ(1-4)GlcNAcβ(1-3)Galβ(1-4)GlcNAcβ(1-3)Galβ(1-4)GlcNAcβ(1-2)Manα(1-6)]-Manβ(1-4)GlcNAcβ(1-4)GlcNAcβ-Asn-NH2 | 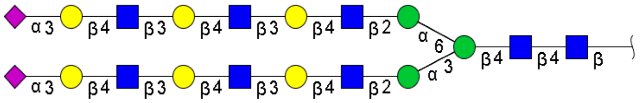 |
| **55** | NeuAcα(2-3)Galβ(1-4)GlcNAcβ(1-3)Galβ(1-4)GlcNAcβ(1-2)Manα(1-3)[NeuAcα(2-3)Galβ(1-4)GlcNAcβ(1-3)Galβ(1-4)GlcNAcβ(1-2)Manα(1-6)]-Manβ(1-4)GlcNAcβ(1-4)GlcNAcβ-(Lys-Val-Ala)Asn-Lys-Thr-NH2 | 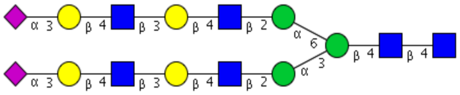 |
| **56** | NeuAcα(2-3)Galβ(1-4)GlcNAcβ(1-3)Galβ(1-4)GlcNAcβ(1-3)Galβ(1-4)GlcNAcβ(1-2)Manα(1-3)[NeuAcα(2-3)Galβ(1-4)GlcNAcβ(1-3)Galβ(1-4)GlcNAcβ(1-3)Galβ(1-4)GlcNAcβ(1-2)Manα(1-6)]-Manβ(1-4)GlcNAcβ(1-4)GlcNAcβ-(Lys-Val-Ala)Asn-Lys-Thr-NH2 | 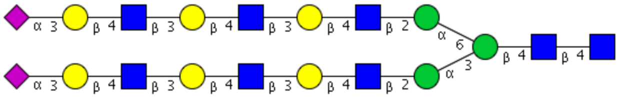 |
| **57** | NeuAcα(2-3)Galβ(1-4)GlcNAcβ(1-3)Galβ(1-4)GlcNAcβ(1-3)Galβ(1-4)GlcNAcβ(1-3)Galβ(1-4)GlcNAcβ(1-2)Manα(1-3)[NeuAcα(2-3)Galβ(1-4)GlcNAcβ(1-3)Galβ(1-4)GlcNAcβ(1-3)Galβ(1-4)GlcNAcβ(1-3)Galβ(1-4)GlcNAcβ(1-2)Manα(1-6)]-Manβ(1-4)GlcNAcβ(1-4)GlcNAcβ-(Lys-Val-Ala)Asn-Lys-Thr-NH2 | 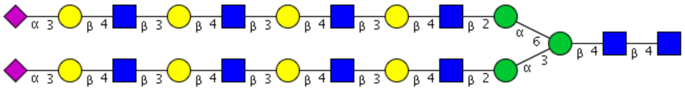 |

| Glycan # | Common Name | Structure |
| --- | --- | --- |
| **58** | NeuAcα(2-3)Galβ(1-4)GlcNAcβ(1-3)Galβ(1-4)GlcNAcβ(1-3)Galβ(1-4)GlcNAcβ(1-3)Galβ(1-4)GlcNAcβ(1-3)Galβ(1-4)GlcNAcβ(1-2)Manα(1-3)[NeuAcα(2-3)Galβ(1-4)GlcNAcβ(1-3)Galβ(1-4)GlcNAcβ(1-3)Galβ(1-4)GlcNAcβ(1-3)Galβ(1-4)GlcNAcβ(1-3)Galβ(1-4)GlcNAcβ(1-2)Manα(1-6)]-Manβ(1-4)GlcNAcβ(1-4)GlcNAcβ-(Lys-Val-Ala)Asn-Lys-Thr-NH2 | 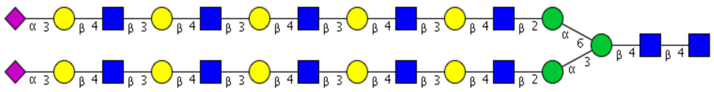 |
| **59** | NeuAcα(2-3)Galβ(1-4)GlcNAcβ(1-3)Galβ(1-4)GlcNAcβ(1-3)Galβ(1-4)GlcNAcβ(1-2)Manα(1-3)[NeuAcα(2-3)Galβ(1-4)GlcNAcβ(1-3)Galβ(1-4)GlcNAcβ(1-3)Galβ(1-4)GlcNAcβ(1-2)Manα(1-6)]-Manβ(1-4)GlcNAcβ(1-4)[Fucα(1-6)]-GlcNAcβ-(Lys-Val-Ala)Asn-Lys-Thr-NH2 | 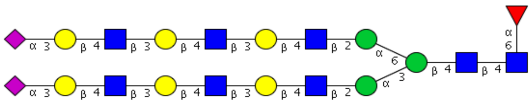 |
| **60** | NeuAcα(2-3)Galβ(1-4)GlcNAcβ(1-3)Galβ(1-4)GlcNAcβ(1-3)Galβ(1-4)GlcNAcβ(1-3)Galβ(1-4)GlcNAcβ(1-2)Manα(1-3)[NeuAcα(2-3)Galβ(1-4)GlcNAcβ(1-3)Galβ(1-4)GlcNAcβ(1-3)Galβ(1-4)GlcNAcβ(1-3)Galβ(1-4)GlcNAcβ(1-2)Manα(1-6)]-Manβ(1-4)GlcNAcβ(1-4)[Fucα(1-6)]-GlcNAcβ-(Lys-Val-Ala)Asn-Lys-Thr-NH2 | 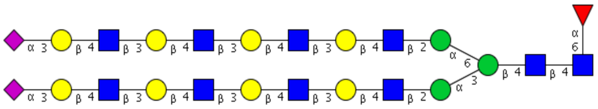 |
| **61** | NeuAcα(2-3)Galβ(1-4)GlcNAcβ(1-3)Galβ(1-4)GlcNAcβ(1-2)Manα(1-3){NeuAcα(2-3)Galβ(1-4)GlcNAcβ(1-3)Galβ(1-4)GlcNAcβ(1-2)[NeuAcα(2-3)Galβ(1-4)GlcNAcβ(1-3)Galβ(1-4)GlcNAcβ(1-6)Manα(1-6)]}-Manβ(1-4)GlcNAcβ(1-4)GlcNAcβ-(Lys-Val-Ala)Asn-Lys-Thr-NH2 | 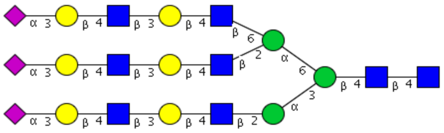 |
| **62** | NeuAcα(2-3)Galβ(1-4)GlcNAcβ(1-3)Galβ(1-4)GlcNAcβ(1-3)Galβ(1-4)GlcNAcβ(1-2)Manα(1-3){NeuAcα(2-3)Galβ(1-4)GlcNAcβ(1-3)Galβ(1-4)GlcNAcβ(1-3)Galβ(1-4)GlcNAcβ(1-2)[NeuAcα(2-3)Galβ(1-4)GlcNAcβ(1-3)Galβ(1-4)GlcNAcβ(1-3)Galβ(1-4)GlcNAcβ(1-6)Manα(1-6)]}-Manβ(1-4)GlcNAcβ(1-4)GlcNAcβ-(Lys-Val-Ala)Asn-Lys-Thr-NH2 | 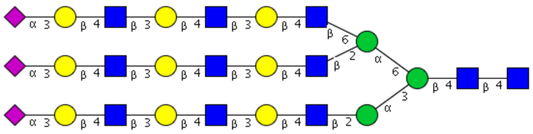 |

| Glycan # | Common Name | Structure |
| --- | --- | --- |
| 63 | NeuAcα(2-3)Galβ(1-4)GlcNAcβ(1-3)Galβ(1-4)GlcNAcβ(1-3)Galβ(1-4)GlcNAcβ(1-3)Galβ(1-4)GlcNAcβ(1-2)Manα(1-3){NeuAcα(2-3)Galβ(1-4)GlcNAcβ(1-3)Galβ(1-4)GlcNAcβ(1-3)Galβ(1-4)GlcNAcβ(1-3)Galβ(1-4)GlcNAcβ(1-2)[NeuAcα(2-3)Galβ(1-4)GlcNAcβ(1-3)Galβ(1-4)GlcNAcβ(1-3)Galβ(1-4)GlcNAcβ(1-3)Galβ(1-4)GlcNAcβ(1-6)Manα(1-6)]}-Manβ(1-4)GlcNAcβ(1-4)GlcNAcβ-(Lys-Val-Ala)Asn-Lys-Thr-NH2 | 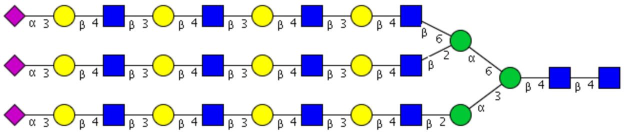 |
| 64 | NeuAcα(2-3)Galβ(1-4)GlcNAcβ(1-3)Galβ(1-4)GlcNAcβ(1-2)Manα(1-3){NeuAcα(2-3)Galβ(1-4)GlcNAcβ(1-3)Galβ(1-4)GlcNAcβ(1-2)[NeuAcα(2-3)Galβ(1-4)GlcNAcβ(1-3)Galβ(1-4)GlcNAcβ(1-6)Manα(1-6)]}-Manβ(1-4)GlcNAcβ(1-4)[Fucα(1-6)]-GlcNAcβ-(Lys-Val-Ala)Asn-Lys-Thr-NH2 | 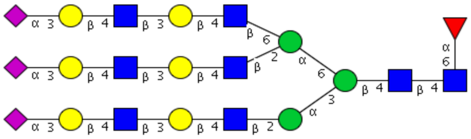 |
| 65 | NeuAcα(2-3)Galβ(1-4)GlcNAcβ(1-3)Galβ(1-4)GlcNAcβ(1-3)Galβ(1-4)GlcNAcβ(1-2)Manα(1-3){NeuAcα(2-3)Galβ(1-4)GlcNAcβ(1-3)Galβ(1-4)GlcNAcβ(1-3)Galβ(1-4)GlcNAcβ(1-2)[NeuAcα(2-3)Galβ(1-4)GlcNAcβ(1-3)Galβ(1-4)GlcNAcβ(1-3)Galβ(1-4)GlcNAcβ(1-6)Manα(1-6)]}-Manβ(1-4)GlcNAcβ(1-4)[Fucα(1-6)]-GlcNAcβ-(Lys-Val-Ala)Asn-Lys-Thr-NH2 | 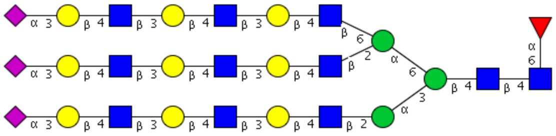 |
| 66 | Gn/3'SLN/3'SLN-TriN | 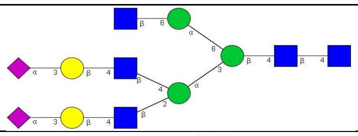 |
| 67 | NeuAcα(2-3)[GalNAcβ(1-4)]-Galβ(1-4)GlcNAcβ-ethyl-NH2 | 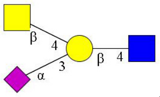 |
| 68 | NeuAcα(2-3)[GalNAcβ(1-4)]-Galβ(1-4)Glcβ-ethyl-NH2 | 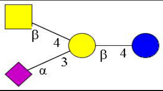 |
| 69 | Galβ(1-3)GalNAcβ(1-4)[NeuAcα(2-3)]-Galβ(1-4)Glcβ-ethyl-NH2 | 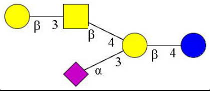 |
| 70 | NeuAcα(2-3)Galβ(1-4)[Fucα(1-3)]-GlcNAcβ-propyl-NH2 | 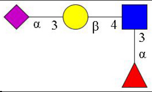 |
| 71 | NeuAcα(2-3)Galβ(1-3)[Fucα(1-4)]-GlcNAcβ(1-3)Galβ(1-4)[Fucα(1-3)]-GlcNAcβ-ethyl-NH2 | 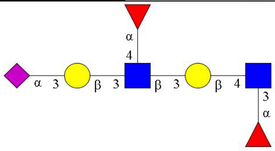 |
| 72 | NeuAcα(2-3)Galβ(1-4)[Fucα(1-3)]-GlcNAcβ(1-3)Galβ(1-4)[Fucα(1-3)]-GlcNAcβ-ethyl-NH2 | 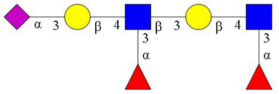 |

| Glycan # | Common Name | Structure |
|---|---|---|
| 73 | NeuAcα(2-3)Galβ(1-4)[Fucα(1-3)]-GlcNAcβ(1-3)Galβ(1-4)[Fucα(1-3)]-GlcNAcβ(1-3)Galβ(1-4)[Fucα(1-3)]-GlcNAcβ-ethyl-NH2 | 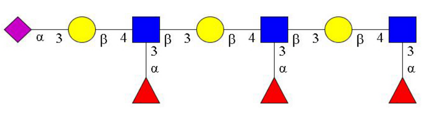 |
| 74 | NeuAcα(2-3)Galβ(1-4)[Fucα(1-3)]-GlcNAcβ(1-3)Galβ(1-4)[Fucα(1-3)]-GlcNAcβ(1-3)Galβ(1-4)[Fucα(1-3)]-GlcNAcβ(1-3)Galβ(1-3)GalNAcα-Thr-NH2 | 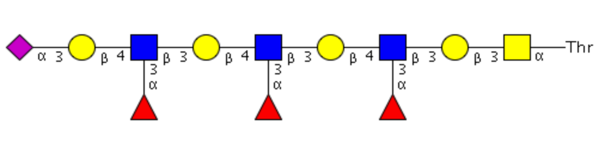  |
| 75 | NeuAcα(2-3)Galβ(1-4)[Fucα(1-3)]-GlcNAcβ(1-3)Galβ(1-4)[Fucα(1-3)]-GlcNAcβ(1-3)Galβ(1-4)[Fucα(1-3)]-GlcNAcβ(1-3)GalNAcα-Thr-NH2 | 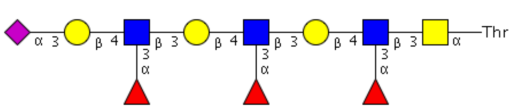  |
| 76 | NeuAcα(2-3)Galβ(1-4)[Fucα(1-3)]-GlcNAcβ(1-3)Galβ(1-4)[Fucα(1-3)]-GlcNAcβ(1-3)Galβ(1-4)[Fucα(1-3)]-GlcNAcβ(1-3)[NeuAcα(2-3)Galβ(1-4)[Fucα(1-3)]-GlcNAcβ(1-3)Galβ(1-4)[Fucα(1-3)]-GlcNAcβ(1-3)Galβ(1-4)[Fucα(1-3)]-GlcNAcβ(1-6)]-GalNAcα-Thr-NH2 | 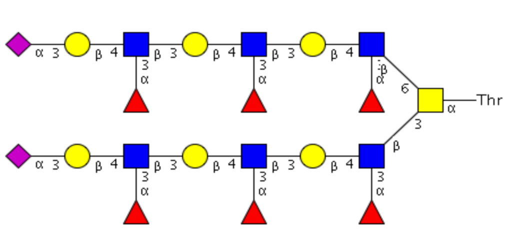  |
| 77 | NeuAcα(2-3)Galβ(1-4)[Fucα(1-3)]-GlcNAcβ(1-2)Manα(1-3)[NeuAcα(2-3)Galβ(1-4)[Fucα(1-3)]-GlcNAcβ(1-2)Manα(1-6)]-Manβ(1-4)GlcNAcβ(1-4)GlcNAcβ-(Lys-Val-Ala)Asn-(Lys-Thr)NH2 | 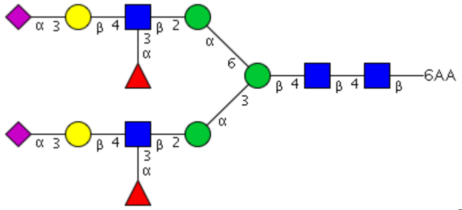  |
| 78 | NeuAcα(2-6)Galb(1-4)(6S)GlcNacb-ethyl-NH2 | 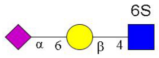  |
| 79 | NeuAcα(2-6)Galβ(1-4)6-O-sulfo-GlcNAcβ-propyl-NH2 | 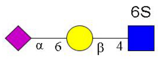  |
| 80 | NeuAcα(2-6)Galβ(1-4)Glcβ-ethyl-NH2 | 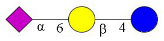 |
| 81 | NeuAcα(2-6)Galβ(1-4)GlcNAcβ-ethyl-NH2 | 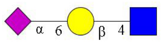 |
| 82 | NeuAcα(2-6)Galβ(1-4)GlcNAcβ(1-3)Galβ(1-4)GlcNAcβ-ethyl-NH2 | 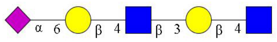 |
| 83 | NeuAcα(2-6)Galβ(1-4)GlcNAcβ(1-3)Galβ(1-4)GlcNAcβ(1-3)Galβ(1-4)GlcNAcβ-ethyl-NH2 | 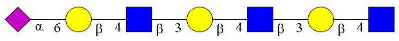 |
| 84 | NeuAcα(2-6)GalNAcβ(1-4)GlcNAcβ-ethyl-NH2 | 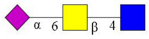 |
| 85 | NeuAcα(2-6)Galβ(1-4)GlcNAcβ(1-3)Galβ(1-3)GalNAcα-Thr-NH2 | 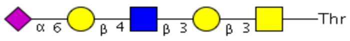  |
| 86 | NeuAcα(2-6)Galβ(1-4)GlcNAcβ(1-3)Galβ(1-4)GlcNAcβ(1-3)Galβ(1-3)GalNAcα-Thr-NH2 | 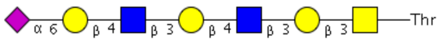  |
| 87 | NeuAcα(2-6)Galβ(1-4)GlcNAcβ(1-3)Galβ(1-4)GlcNAcβ(1-3)Galβ(1-4)GlcNAcβ(1-3)Galβ(1-3)GalNAcα-Thr-NH2 | 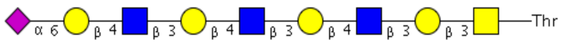  |
| 88 | NeuAcα(2-6)Galβ(1-4)GlcNAcβ(1-3)Galβ(1-4)GlcNAcβ(1-3)Galβ(1-4)GlcNAcβ(1-3)Galβ(1-4)GlcNAcβ(1-3)Galβ(1-3)GalNAcα-Thr-NH2 | 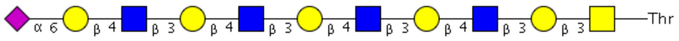  |

| Glycan # | Common Name | Structure |
|---|---|---|
| 89 | NeuAcα(2-6)Galβ(1-4)GlcNAcβ(1-3)Galβ(1-4)GlcNAcβ(1-3)Galβ(1-4)GlcNAcβ(1-3)Galβ(1-4)GlcNAcβ(1-3)Galβ(1-4)GlcNAcβ(1-3)Galβ(1-3)GalNAcα-Thr-NH2 | 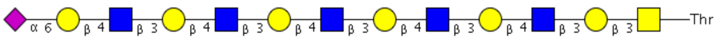 |
| 90 | NeuAcα(2-6)Galβ(1-4)GlcNAcβ(1-6)[Galβ(1-3)]-GalNAcα-Thr-NH2 | 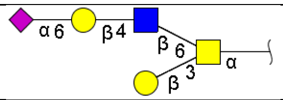 |
| 91 | NeuAcα(2-6)Galβ(1-4)GlcNAcβ(1-3)Galβ(1-4)GlcNAcβ(1-6)[Galβ(1-3)]-GalNAcα-Thr-NH2 | 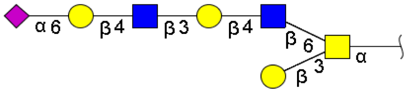 |
| 92 | NeuAcα(2-6)Galβ(1-4)GlcNAcβ(1-3)Galβ(1-4)GlcNAcβ(1-3)Galβ(1-4)GlcNAcβ(1-6)[Galβ(1-3)]-GalNAcα-Thr-NH2 | 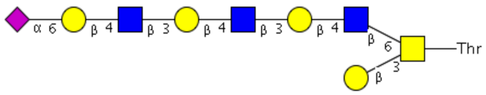 |
| 93 | NeuAcα(2-6)Galβ(1-4)GlcNAcβ(1-3)Galβ(1-4)GlcNAcβ(1-3)Galβ(1-4)GlcNAcβ(1-3)Galβ(1-4)GlcNAcβ(1-6)[Galβ(1-3)]-GalNAcα-Thr-NH2 | 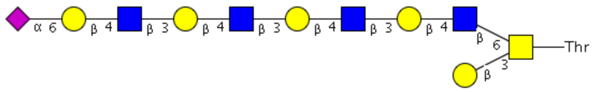 |
| 94 | NeuAcα(2-6)Galβ(1-4)GlcNAcβ(1-3)Galβ(1-4)GlcNAcβ(1-3)Galβ(1-4)GlcNAcβ(1-3)Galβ(1-4)GlcNAcβ(1-3)Galβ(1-4)GlcNAcβ(1-6)[Galβ(1-3)]-GalNAcα-Thr-NH2 | 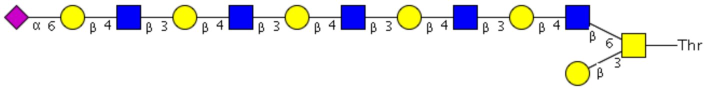 |
| 95 | NeuAcα(2-6)Galβ(1-4)GlcNAcβ(1-3)Galβ(1-4)GlcNAcβ(1-3)Galβ(1-4)GlcNAcβ(1-3)Galβ(1-4)GlcNAcβ(1-6)[NeuAcα(2-6)Galβ(1-4)GlcNAcβ(1-3)Galβ(1-4)GlcNAcβ(1-3)Galβ(1-4)GlcNAcβ(1-3)Galβ(1-3)]-GalNAcα-Thr-NH2 | 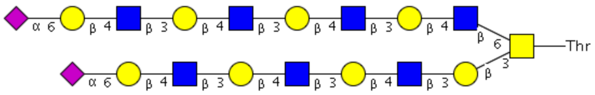 |
| 96 | NeuAcα(2-6)Galβ(1-4)GlcNAcβ(1-3)Galβ(1-4)GlcNAcβ(1-3)Galβ(1-4)GlcNAcβ(1-3)Galβ(1-4)GlcNAcβ(1-3)Galβ(1-4)GlcNAcβ(1-6)[NeuAcα(2-6)Galβ(1-4)GlcNAcβ(1-3)Galβ(1-4)GlcNAcβ(1-3)Galβ(1-4)GlcNAcβ(1-3)Galβ(1-4)GlcNAcβ(1-3)Galβ(1-3)]-GalNAcα-Thr-NH2 | 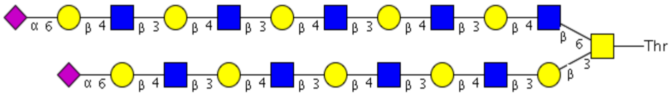 |
| 97 | NeuAcα(2-6)Galβ(1-4)GlcNAcβ(1-3)GalNAcα-Thr-NH2 | 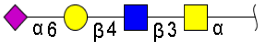 |
| 98 | NeuAcα(2-6)Galβ(1-4)GlcNAcβ(1-3)Galβ(1-4)GlcNAcβ(1-3)GalNAcα-Thr-NH2 | 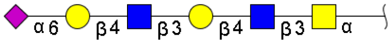 |
| 99 | NeuAcα(2-6)Galβ(1-4)GlcNAcβ(1-3)Galβ(1-4)GlcNAcβ(1-3)Galβ(1-4)GlcNAcβ(1-3)GalNAcα-Thr-NH2 | 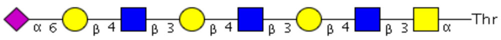 |
| 100 | NeuAcα(2-6)Galβ(1-4)GlcNAcβ(1-3)Galβ(1-4)GlcNAcβ(1-3)Galβ(1-4)GlcNAcβ(1-3)Galβ(1-4)GlcNAcβ(1-3)GalNAcα-Thr-NH2 | 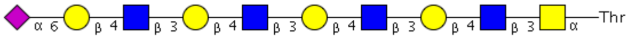 |
| 101 | NeuAcα(2-6)Galβ(1-4)GlcNAcβ(1-3)Galβ(1-4)GlcNAcβ(1-3)Galβ(1-4)GlcNAcβ(1-3)Galβ(1-4)GlcNAcβ(1-3)Galβ(1-4)GlcNAcβ(1-3)GalNAcα-Thr-NH2 | 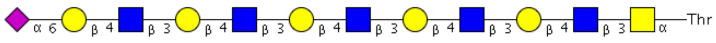 |

| Glycan # | Common Name | Structure |
|---|---|---|
| 102 | NeuAcα(2-6)Galβ(1-4)GlcNAcβ(1-3)[NeuAcα(2-6)Galβ(1-4)GlcNAcβ(1-6)]-GalNAcα-Thr-NH2 | 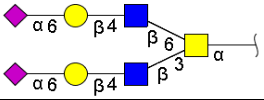 |
| 103 | NeuAcα(2-6)Galβ(1-4)GlcNAcβ(1-3)Galβ(1-4)GlcNAcβ(1-3)[NeuAcα(2-6)Galβ(1-4)GlcNAcβ(1-3)Galβ(1-4)GlcNAcβ(1-6)]-GalNAcα-Thr-NH2 | 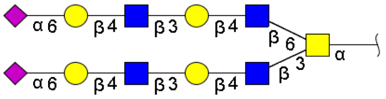 |
| 104 | NeuAcα(2-6)Galβ(1-4)GlcNAcβ(1-3)Galβ(1-4)GlcNAcβ(1-3)Galβ(1-4)GlcNAcβ(1-3)[NeuAcα(2-6)Galβ(1-4)GlcNAcβ(1-3)Galβ(1-4)GlcNAcβ(1-3)Galβ(1-4)GlcNAcβ(1-6)]-GalNAcα-Thr-NH2 | 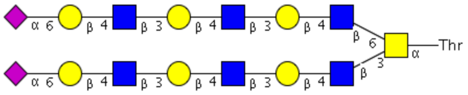 |
| 105 | NeuAcα(2-6)Galβ(1-4)GlcNAcβ(1-3)Galβ(1-4)GlcNAcβ(1-3)Galβ(1-4)GlcNAcβ(1-3)Galβ(1-4)GlcNAcβ(1-3)[NeuAcα(2-6)Galβ(1-4)GlcNAcβ(1-3)Galβ(1-4)GlcNAcβ(1-3)Galβ(1-4)GlcNAcβ(1-3)Galβ(1-4)GlcNAcβ(1-6)]-GalNAcα-Thr-NH2 | 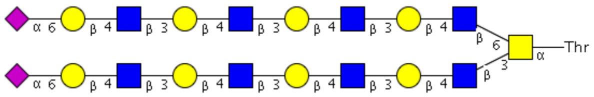 |
| 106 | NeuAcα(2-6)Galβ(1-4)GlcNAcβ(1-3)Galβ(1-4)GlcNAcβ(1-3)Galβ(1-4)GlcNAcβ(1-3)Galβ(1-4)GlcNAcβ(1-3)Galβ(1-4)GlcNAcβ(1-3)[NeuAcα(2-6)Galβ(1-4)GlcNAcβ(1-3)Galβ(1-4)GlcNAcβ(1-3)Galβ(1-4)GlcNAcβ(1-3)Galβ(1-4)GlcNAcβ(1-3)Galβ(1-4)GlcNAcβ(1-6)]-GalNAcα-Thr-NH2 | 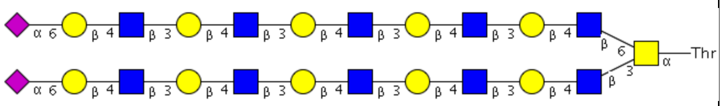 |
| 107 | NeuAcα(2-6)Galβ(1-4)GlcNAcβ(1-3)Galβ(1-4)GlcNAcβ(1-3)Galβ(1-4)GlcNAcβ(1-3)Galβ(1-4)GlcNAcβ(1-6)GalNAcα-Thr-NH2 | 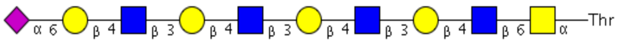 |
| 108 | NeuAcα(2-6)Galβ(1-4)GlcNAcβ(1-3)Galβ(1-4)GlcNAcβ(1-3)Galβ(1-4)GlcNAcβ(1-3)Galβ(1-4)GlcNAcβ(1-3)Galβ(1-4)GlcNAcβ(1-6)GalNAcα-Thr-NH2 | 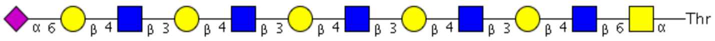 |
| 109 | NeuAcα(2-6)Galβ(1-4)GlcNAcβ(1-3)Galβ(1-4)GlcNAcβ(1-3)Galβ(1-4)GlcNAcβ(1-6)[NeuAcα(2-6)Galβ(1-4)GlcNAcβ(1-3)Galβ(1-4)GlcNAcβ(1-3)] Galβ(1-4)GlcNAcβ-ethyl-NH2 | 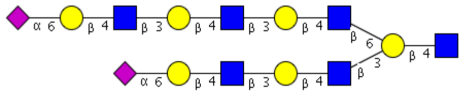 |
| 110 | NeuAcα(2-6)Galβ(1-3)GlcNAcβ(1-3)Galβ(1-4)GlcNAcβ(1-6)[NeuAcα(2-6)Galβ(1-3)GlcNAcβ(1-3)] Galβ(1-4)GlcNAcβ-ethyl-NH2 | 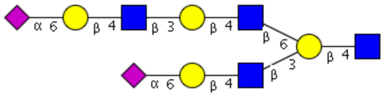 |
| 111 | Galβ(1-4)GlcNAcβ(1-2)Manα(1-3)[NeuAcα(2-6)Galβ(1-4)GlcNAcβ(1-2)Manα(1-6)]-Manβ(1-4)GlcNAcβ(1-4)GlcNAcβ-Asn-NH2 | 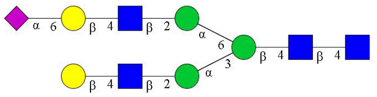 |
| 112 | NeuAcα(2-6)Galβ(1-4)GlcNAcβ(1-2)Manα(1-3)[Galβ(1-4)GlcNAcβ(1-2)Manα(1-6)]-Manβ(1-4)GlcNAcβ(1-4)GlcNAcβ-Asn-NH2 | 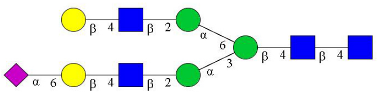 |
| 113 | GlcNAcβ(1-2)Manα(1-3)[NeuAcα(2-6)Galβ(1-4)GlcNAcβ(1-2)Manα(1-6)]-Manβ(1-4)GlcNAcβ(1-4)GlcNAcβ-Asn-NH2 | 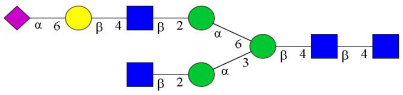 |

| Glycan # | Common Name | Structure |
|---|---|---|
| **114** | NeuAcα(2-6)Galβ(1-4)GlcNAcβ(1-2)Manα(1-3)[NeuAcα(2-6)Galβ(1-4)GlcNAcβ(1-2)Manα(1-6)]-Manβ(1-4)GlcNAcβ(1-4)GlcNAcβ-Asn-NH2 | 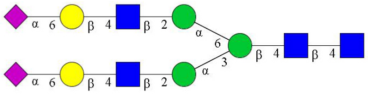 |
| **115** | NeuAcα(2-6)Galβ(1-4)GlcNAcβ(1-3)Galβ(1-4)GlcNAcβ(1-2)Manα(1-3)[NeuAcα(2-6)Galβ(1-4)GlcNAcβ(1-3)Galβ(1-4)GlcNAcβ(1-2)Manα(1-6)]-Manβ(1-4)GlcNAcβ(1-4)GlcNAcβ-Asn-NH2 | 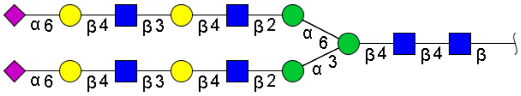 |
| **116** | NeuAcα(2-6)Galβ(1-4)GlcNAcβ(1-3)Galβ(1-4)GlcNAcβ(1-2)Manα(1-3)[NeuAcα(2-6)Galβ(1-4)GlcNAcβ(1-3)Galβ(1-4)GlcNAcβ(1-2)Manα(1-6)]-Manβ(1-4)GlcNAcβ(1-4)GlcNAcβ-(Lys-Val-Ala)Asn-Lys-Thr-NH2 | 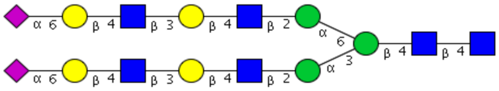 |
| **117** | NeuAcα(2-6)Galβ(1-4)GlcNAcβ(1-3)Galβ(1-4)GlcNAcβ(1-3)Galβ(1-4)GlcNAcβ(1-2)Manα(1-3)[NeuAcα(2-6)Galβ(1-4)GlcNAcβ(1-3)Galβ(1-4)GlcNAcβ(1-3)Galβ(1-4)GlcNAcβ(1-2)Manα(1-6)]-Manβ(1-4)GlcNAcβ(1-4)GlcNAcβ-Asn-NH2 | 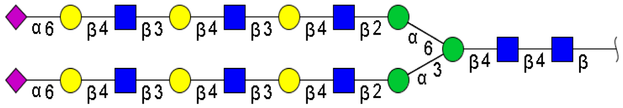 |
| **118** | NeuAcα(2-6)Galβ(1-4)GlcNAcβ(1-3)Galβ(1-4)GlcNAcβ(1-3)Galβ(1-4)GlcNAcβ(1-2)Manα(1-3)[NeuAcα(2-6)Galβ(1-4)GlcNAcβ(1-3)Galβ(1-4)GlcNAcβ(1-3)Galβ(1-4)GlcNAcβ(1-2)Manα(1-6)]-Manβ(1-4)GlcNAcβ(1-4)GlcNAcβ-(Lys-Val-Ala)Asn-Lys-Thr-NH2 | 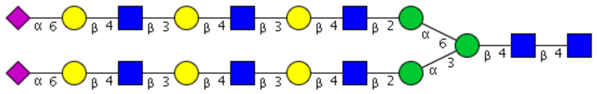 |
| **119** | NeuAcα(2-6)Galβ(1-4)GlcNAcβ(1-3)Galβ(1-4)GlcNAcβ(1-3)Galβ(1-4)GlcNAcβ(1-3)Galβ(1-4)GlcNAcβ(1-2)Manα(1-3)[NeuAcα(2-6)Galβ(1-4)GlcNAcβ(1-3)Galβ(1-4)GlcNAcβ(1-3)Galβ(1-4)GlcNAcβ(1-3)Galβ(1-4)GlcNAcβ(1-2)Manα(1-6)]-Manβ(1-4)GlcNAcβ(1-4)GlcNAcβ-(Lys-Val-Ala)Asn-Lys-Thr-NH2 | 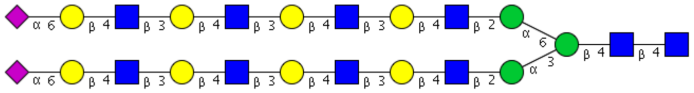 |
| **120** | NeuAcα(2-6)Galβ(1-4)GlcNAcβ(1-3)Galβ(1-4)GlcNAcβ(1-3)Galβ(1-4)GlcNAcβ(1-3)Galβ(1-4)GlcNAcβ(1-3)Galβ(1-4)GlcNAcβ(1-2)Manα(1-3)[NeuAcα(2-6)Galβ(1-4)GlcNAcβ(1-3)Galβ(1-4)GlcNAcβ(1-3)Galβ(1-4)GlcNAcβ(1-3)Galβ(1-4)GlcNAcβ(1-3)Galβ(1-4)GlcNAcβ(1-2)Manα(1-6)]-Manβ(1-4)GlcNAcβ(1-4)GlcNAcβ-(Lys-Val-Ala)Asn-Lys-Thr-NH2 | 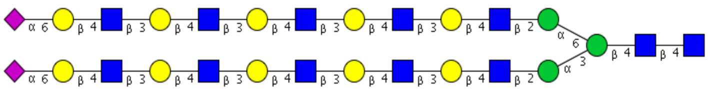 |

| Glycan # | Common Name | Structure |
|---|---|---|
| **121** | NeuAcα(2-6)Galβ(1-4)GlcNAcβ(1-3)Galβ(1-4)GlcNAcβ(1-2)Manα(1-3)[NeuAcα(2-6)Galβ(1-4)GlcNAcβ(1-3)Galβ(1-4)GlcNAcβ(1-2)Manα(1-6)]-Manβ(1-4)GlcNAcβ(1-4)[Fucα(1-6)]-GlcNAcβ-(Lys-Val-Ala)Asn-Lys-Thr-NH2 | 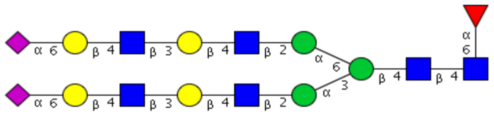 |
| **122** | NeuAcα(2-6)Galβ(1-4)GlcNAcβ(1-3)Galβ(1-4)GlcNAcβ(1-3)Galβ(1-4)GlcNAcβ(1-2)Manα(1-3)[NeuAcα(2-6)Galβ(1-4)GlcNAcβ(1-3)Galβ(1-4)GlcNAcβ(1-3)Galβ(1-4)GlcNAcβ(1-2)Manα(1-6)]-Manβ(1-4)GlcNAcβ(1-4)[Fucα(1-6)]-GlcNAcβ-(Lys-Val-Ala)Asn-Lys-Thr-NH2 | 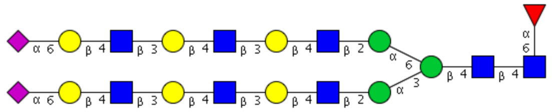 |
| **123** | NeuAcα(2-6)Galβ(1-4)GlcNAcβ(1-3)Galβ(1-4)GlcNAcβ(1-3)Galβ(1-4)GlcNAcβ(1-3)Galβ(1-4)GlcNAcβ(1-2)Manα(1-3)[NeuAcα(2-6)Galβ(1-4)GlcNAcβ(1-3)Galβ(1-4)GlcNAcβ(1-3)Galβ(1-4)GlcNAcβ(1-3)Galβ(1-4)GlcNAcβ(1-2)Manα(1-6)]-Manβ(1-4)GlcNAcβ(1-4)[Fucα(1-6)]-GlcNAcβ-(Lys-Val-Ala)Asn-Lys-Thr-NH2 | 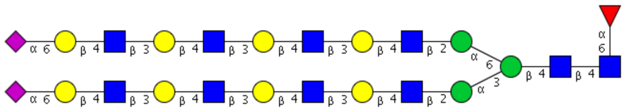 |
| **124** | NeuAcα(2-6)Galβ(1-4)GlcNAcβ(1-3)Galβ(1-4)GlcNAcβ(1-2)Manα(1-3){NeuAcα(2-6)Galβ(1-4)GlcNAcβ(1-3)Galβ(1-4)GlcNAcβ(1-2)[NeuAcα(2-6)Galβ(1-4)GlcNAcβ(1-3)Galβ(1-4)GlcNAcβ(1-6)Manα(1-6)]}-Manβ(1-4)GlcNAcβ(1-4)GlcNAcβ-(Lys-Val-Ala)Asn-Lys-Thr-NH2 | 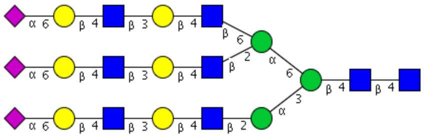 |
| **125** | NeuAcα(2-6)Galβ(1-4)GlcNAcβ(1-3)Galβ(1-4)GlcNAcβ(1-2)Manα(1-3){NeuAcα(2-6)Galβ(1-4)GlcNAcβ(1-3)Galβ(1-4)GlcNAcβ(1-2)[NeuAcα(2-6)Galβ(1-4)GlcNAcβ(1-3)Galβ(1-4)GlcNAcβ(1-6)Manα(1-6)]}-Manβ(1-4)GlcNAcβ(1-4)[Fucα(1-6)]-GlcNAcβ-(Lys-Val-Ala)Asn-Lys-Thr-NH2 | 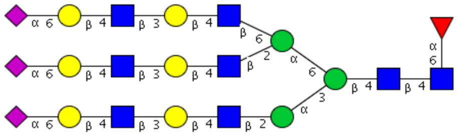 |
| **126** | NeuAcα(2-6)Galβ(1-4)GlcNAcβ(1-3)Galβ(1-4)GlcNAcβ(1-3)Galβ(1-4)GlcNAcβ(1-2)Manα(1-3){NeuAcα(2-6)Galβ(1-4)GlcNAcβ(1-3)Galβ(1-4)GlcNAcβ(1-3)Galβ(1-4)GlcNAcβ(1-2)[NeuAcα(2-6)Galβ(1-4)GlcNAcβ(1-3)Galβ(1-4)GlcNAcβ(1-3)Galβ(1-4)GlcNAcβ(1-6)Manα(1-6)]}-Manβ(1-4)GlcNAcβ(1-4)[Fucα(1-6)]-GlcNAcβ-(Lys-Val-Ala)Asn-Lys-Thr-NH2 | 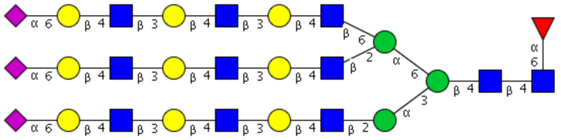 |

| Glycan # | Common Name | Structure |
|---|---|---|
| **127** | NeuAcα(2-6)Galβ(1-4)GlcNAcβ(1-3)Galβ(1-4)GlcNAcβ(1-3)Galβ(1-4)GlcNAcβ(1-3)Galβ(1-4)GlcNAcβ(1-2)Manα(1-3){NeuAcα(2-6)Galβ(1-4)GlcNAcβ(1-3)Galβ(1-4)GlcNAcβ(1-3)Galβ(1-4)GlcNAcβ(1-3)Galβ(1-4)GlcNAcβ(1-2)[NeuAcα(2-6)Galβ(1-4)GlcNAcβ(1-3)Galβ(1-4)GlcNAcβ(1-3)Galβ(1-4)GlcNAcβ(1-3)Galβ(1-4)GlcNAcβ(1-6)Manα(1-6)]}-Manβ(1-4)GlcNAcβ(1-4)[Fucα(1-6)]-GlcNAcβ-(Lys-Val-Ala)Asn-Lys-Thr-NH2 | 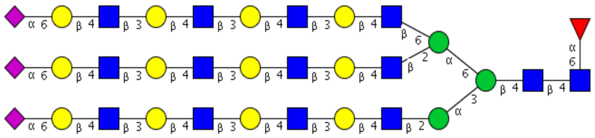 |
| **128** | LN/6'SLN/6'SLN-TriN | 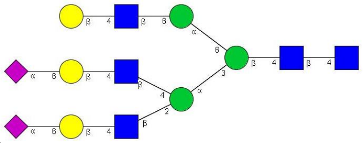 |
| **129** | 6'SLN/LeX/LeX-TriN | 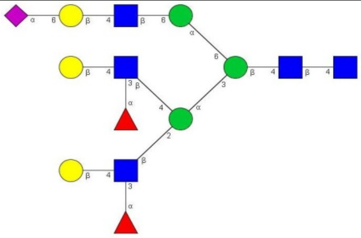 |
| **130** | 6'SLNLN/LeX/LeX-TriN | 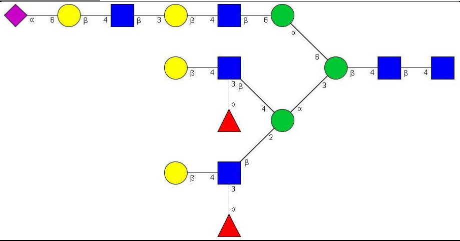 |

**Supplementary References**


1. Stothard P. The sequence manipulation suite: JavaScript programs for analyzing and formatting protein and DNA sequences. *Biotechniques* **28**, 1102, 1104 (2000).

2. Ekiert DC*, et al.* Cross-neutralization of influenza A viruses mediated by a single antibody loop. *Nature* **489**, 526-532 (2012).

3. Lin YP*, et al.* Evolution of the receptor binding properties of the influenza A(H3N2) hemagglutinin. *Proc Natl Acad Sci U S A* **109**, 21474-21479 (2012).

4. Lee PS*, et al.* Receptor mimicry by antibody F045-092 facilitates universal binding to the H3 subtype of influenza virus. *Nat Commun* **5**, 3614 (2014).

5. Das R, Baker D. Macromolecular modeling with rosetta. *Annu Rev Biochem* **77**, 363-382 (2008).

6. Kellogg EH, Leaver-Fay A, Baker D. Role of conformational sampling in computing mutation-induced changes in protein structure and stability. *Proteins* **79**, 830-838 (2011).
