# Supplementary material for: A complex epistatic network limits the mutational reversibility in the influenza hemagglutinin receptor-binding site
Source: Nat Commun. 2018 Mar 28;9:1264. doi: 10.1038/s41467-018-03663-5 (PMC5871881; doi:10.1038/s41467-018-03663-5)
Supplement: Supplementary file 3 — Description of Additional Supplementary Files(PDF 3 kb) [file 41467_2018_3663_MOESM3_ESM.pdf]

Description of Additional Supplementary Files

File Name: Supplementary Data 1
Description: RF index for individual variants.
